# Antimicrobial Potential of Secalonic Acids from Arctic-Derived *Penicillium chrysogenum* INA 01369

**DOI:** 10.3390/antibiotics14010088

**Published:** 2025-01-14

**Authors:** Yulia A. Roshka, Natalia N. Markelova, Sofia D. Mashkova, Kseniya V. Malysheva, Marina L. Georgieva, Igor B. Levshin, Vladimir I. Polshakov, Alexander M. Arutyunian, Alexey S. Vasilchenko, Vera S. Sadykova

**Affiliations:** 1 Laboratory for Taxonomic Study and Collection of Cultures of Microorganisms, Gause Institute of New Antibiotics, St. Bolshaya Pirogovskaya, 11, 119021 Moscow, Russia; roshkajulia@gmail.com (Y.A.R.); nathanmrk82@gmail.com (N.N.M.); maskovasofa9@gmail.com (S.D.M.); malishevaks@yandex.ru (K.V.M.); i-marina@yandex.ru (M.L.G.); levshin@panavir.ru (I.B.L.); 2 Faculty of Biology, Lomonosov Moscow State University, 1-12 Leninskie Gory, 119234 Moscow, Russia; 3 Center for Magnetic Tomography & Spectroscopy, Faculty of Fundamental Medicine, Lomonosov Moscow State University, Leninskie Gory, GSP-1, 119991 Moscow, Russia; vpolsha@fbm.msu.ru; 4 A.N. Belozersky Institute of Physico-Chemical Biology, Lomonosov Moscow State University, Leninskie Gory, GSP-1, 119991 Moscow, Russia; alarut@belozersky.msu.ru; 5 Laboratory of Antimicrobial Resistance, Institute of Environmental and Agricultural Biology (X-BIO), Tyumen State University, 625003 Tyumen, Russia; avasilchenko@gmail.com

**Keywords:** psychrotolerant fungi, *Penicillium chrysogenum*, antimicrobial activity, secalonic acid

## Abstract

In this study, two compounds have been isolated from the Arctic-derived fungus *Penicillium chrysogenum* INA 13460. Structural elucidation, performed using 2D NMR and HR-ESIMS data, has identified the compounds as stereoisomers of secalonic acids, dimeric tetrahydroxanthones. The absolute configurations of these stereoisomers have been determined through conformational NMR analysis and circular dichroism spectroscopy. The antimicrobial activity of secalonic acids D and F has been evaluated against a diverse range of microorganisms, including Gram-positive multidrug-resistant *Staphylococcus aureus*, Gram-negative *Escherichia coli* ATCC 25922, *Pseudomonas aeruginosa* ATCC 27853, the phytopathogen *Pectobacterium carotovorum* VKM-B1247, and the fungi *Fusarium oxysporum* VKPM F 890, *Aspergillus fumigatus* VKM F-37, and *A. niger* ATCC 16404. Genomic and chemical analyses further support *P. chrysogenum* INA 13460 as a promising natural source for antimicrobial drug discovery and biological control applications.

## 1. Introduction

*Penicillium* is a diverse genus of fungi that includes over 350 known species that have been described in the scientific literature. It is widely distributed in different parts of the world, including Asia, Europe, and Africa, as well as the coldest places on Earth [1,2,3]. For instance, representatives of *Penicillium* fungi have the potential to thrive in extreme environmental conditions such as low temperature (psychrophiles or psychrotolerant fungi range below 30 °C), high light irradiance, and desiccation. It can be found all over the world on a variety of substrates, such as soil and food, and in a variety of ways, ranging from necrotrophic pathogenicity to endophytic mutualism [4,5,6,7,8,9]. Microscopic fungi of the genus *Penicillium* produce more than 15,000 bioactive compounds useful for human health, more than half of which have antimicrobial potential [5,10,11,12,13,14].

*Penicillium chrysogenum*, a member of the genus *Penicillium* section *Chrysogena*, is an efficient producer of several conventional antibiotics [1,15,16,17,18,19]. Many ß-lactam antibiotics, including penicillin and other variable bioactive compounds with antimicrobial and anticancer properties, have been found to originate from this species. In recent years, a wide variety of natural products have been reported to originate from *P. chrysogenum*, including peptides, alkaloids, polyketides, terpenoids, pyrones, and steroids [1,7,12,13,14,20,21]. Up until 2023, 277 compounds from *P. chrysogenum* had been identified using both conventional and contemporary analytical techniques [2,15,20,21].

This article reports on a chemical investigation of the antibiotic compounds of the psychrotolerant fungus *Penicillium chrysogenum* INA 01369, involving the isolation, structure elucidation, and biological evaluation of compounds. Therefore, the present study aimed to characterize the fungus with a focus on genetic mining and optimizing culture conditions for antibiotic compound production.

## 2. Results

**Figure 1:**
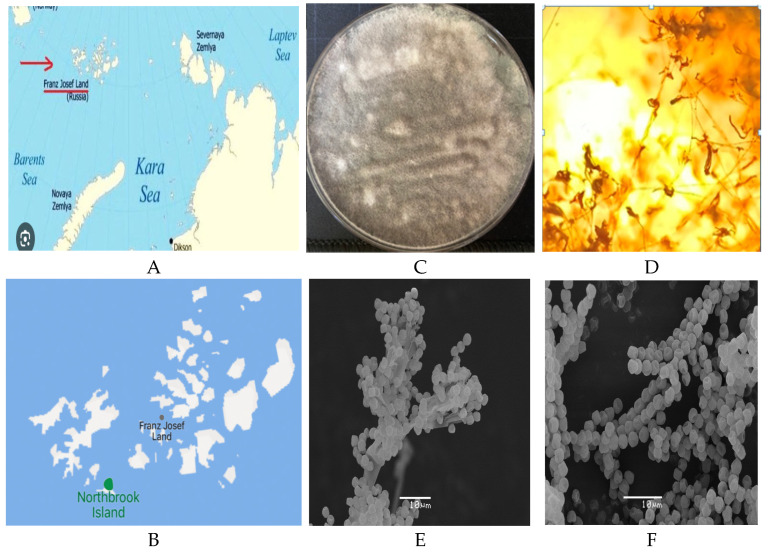
(**A**,**B**) Location of the Northbrook Island; Morphology *P. chrysogenum* INA 01369 after cultivation for seven days on PDA at 20 °C; (**C**) colony on PDA at 20 °C; (**D**) conidiophores and conidial sporulation (LM); (**E**,**F**) conidiophores and conidial sporulation (SEM).

In our ongoing research for the discovery of bioactive secondary metabolites from the psychrotolerant fungi [22], identification and chemical investigation have been performed on a fungal strain INA 01369 of *Penicillium* sp. section *Chrysogena* obtained from the soil of a bird colony on the flat of Northbrook Island (Figure 1A,B). Based on the area of growth in the five different temperatures on two solid media, PDA and Chapek Dox from 5 to 25 °C, the strain can be considered as psychrotolerant [23,24,25]. The strain has shown the best growth at 20 °C on both media and did not grow at temperatures above 28 °C (Supplementary S1: Figure S1).

### 2.1. Identification and Phylogenetic Analysis of Strain INA 01369

Strain INA 01369 exhibited a green and dense furry surface with a white edge and produced yellow-pigmented colonies when cultured on Chapek Dox plates at 20 °C for 5 days. The color of its conidial mass can range from green to grayish green (Figure 1C). Microscopic observation showed transparent, tubular, branched hyphae with septa (Figure 1D), producing brush-like clusters of branching conidiophores, intermediate branches, and globose, smooth spores (6–8 μm diameter) (Figure 1E,F).

These characteristics are consistent with *P. chrysogenum* strain morphology [1]. The strain was identified as *P. chrysogenum* based on morphological features and on phylogenetic analysis.

**Figure 2:**
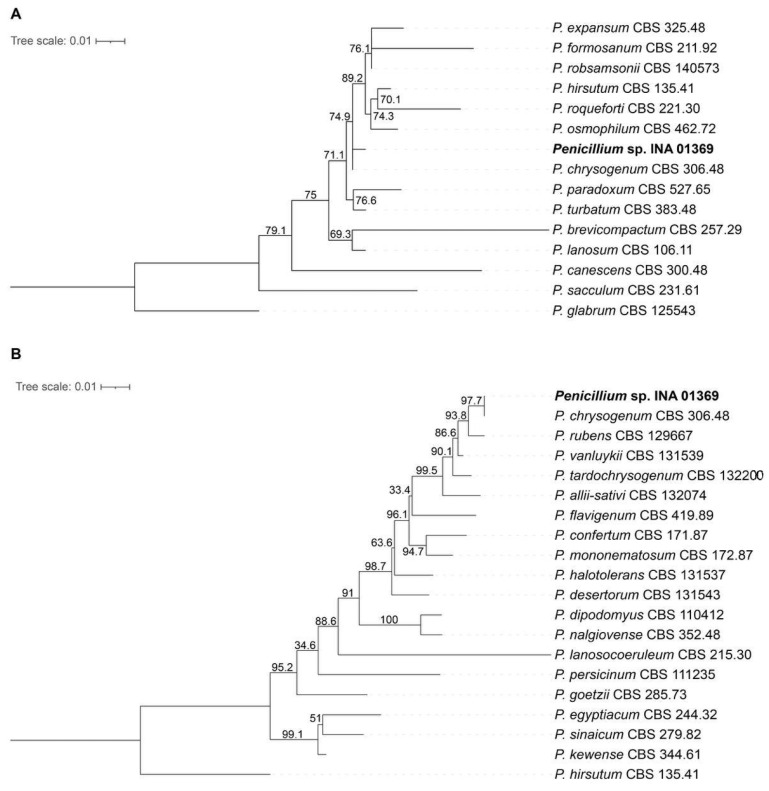
IQ-TREE phylogenetic trees constructed for *Penicillium chrysogenum* INA 01369 sequences. Best-fit models of substitution according to BIC: TIM2e+I+G4 (**A**), TNe+G4 (**A**). The numbers indicate bootstrap values. We specified 1000 iterations for bootstrap. (**A**) Tree constructed for 15 ITS sequences. The phylogram is rooted with *P. glabrum*. (**B**) Tree constructed for 20 sequences represented by three concatenated genes for β-tubulin, calmodulin, and DNA-directed RNA polymerase II core subunit. The phylogram is rooted with *P. hirsutum*. The BP values are displayed on the nodes (BP; 1000 replicates).

A phylogenetic tree has been constructed based on the nucleotide sequence from assemblies of genomes from INA 01369 and other strains, available in the database and listed in Supplementary S1: Table S1. At first, the ITS sequence of strain INA 01369 was aligned with 14 sequences retrieved from GenBank, which included 13 sequences ex-type strains of *Penicillium* species of *Penicillium* subgenus and 1 outgroup taxon *Penicillium glabrum* CBS 125543T (subgen. *Aspergilloides*, sect. *Aspergilloides*) (Figure 2A; Supplementary S1: Table S1).

Then, β-tubulin, calmodulin, and DNA-directed RNA polymerase II core subunit sequences of strain INA 01369 were aligned with 19 sequences retrieved from GenBank, which included 18 sequences ex-type strains of *Penicillium* species of *Chrysogena* section (subgen. *Penicillium*) and 1 outgroup taxon *Penicillium hirsutum* CBS 135.41T (subgen. *Penicillium*, sect. *Fasciculata*, ser. *Corymbifera*) (Figure 2B; Supplementary S1: Table S2). The strain INA 01369 has been grouped together with the ex-type strain of *Penicillium chrysogenum* (CBS 171.87T) in both the ITS-based and three loci (β-tubulin, calmodulin, and DNA-directed RNA polymerase II core subunit) phylogenetic analyses (Figure 2A,B in Supplementary S1: Table S2) and showed high support within this group.

### 2.2. Effects of the Cultivation for Growth, Sporulation, and Secalonic Acid Complex Production

**Figure 3:**
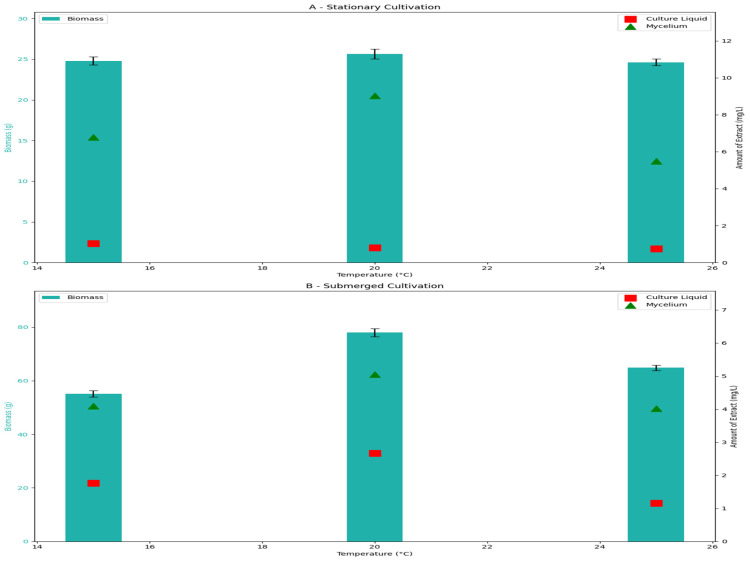
Production of secalonic acids (bars) and biomass (triangle and square) in different cultivation conditions: (**A**) stationary condition; (**B**) submerged condition.

To obtain a global view of the effects of different temperatures and evaluate this ability to produce active secondary metabolites, we compared strain *P. chrysogenum* INA 01369 cultured at 15, 20, and 25 °C on PDB. Formation of the biomass by the producer and the amount of crude active extract has been analyzed under stationary and submerged cultivation (Figure 3).

The fungus showed a gradual increase in biomass over time, indicating its ability to grow under different conditions and temperatures.

However, compared to submerged conditions, the growth of the strain was slower and resulted in lower biomass in the stationary cultivation. INA 01369 was moderately psychrotolerant, with optimal growth at 20 °C and a decreased growth rate when the temperature exceeded 25 °C. Furthermore, a maximal amount of 9.03 mg/L of antibiotic compounds was observed in fungal mycelium when grown in stationary conditions at 20 °C. The maximal observed amount of antibiotic compounds when grown in submerged conditions was 4.09 mg/L. The strain produced a significantly lower amount of antibiotic compounds in culture liquid extract compared to mycelium (Figure 3). Subsequently, the culture broth maintained at 25 °C resulted in a decreased production of antibiotic compounds.

Extraction of the antibiotics in cell-free supernatant and the culture broth has been performed with different organic solvents; however, ethyl acetate was found to be the most suitable among all the solvents tested. Both fractions have been dried and re-dissolved in 50% ethanol. Based on the HPLC chromatographic separation of ethanol concentrates, two active compounds have been obtained and further investigated (Supplementary S1: Figure S2).

### 2.3. Genome Sequencing of Strain INA 01369 and Genome Annotation

The whole genome sequence has been deposited in the NCBI database under the Bioproject number PRJNA1185248 (BioSample ID: SAMN44697790). In total, 486,827 open reading frames were detected with GetORF. Thirty-seven thousand five hundred and forty-seven protein-coding sequences and 10,500 mRNAs were predicted in the final official gene set of genSAS annotation. Augustus detected 37,457 protein-coding sequences and 10,500 mRNAs. Based on Pfam domain characterization, 8897 genes belonged to 3441 protein families and the GC content was 48.84%. The RepeatMasker program identified 7028 repeating regions. DIAMOD detected 10,083 proteins. The tRNAscan-SE program found 214 tRNAs.

**Figure 4:**
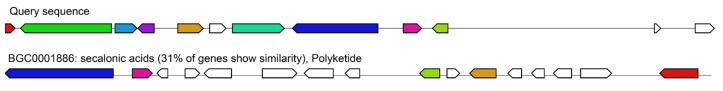
Schematic representation of clusters. Comparison of the found cluster with the known secalonic acid biosynthesis cluster from the MiBIG database. The genes marked with the same color are interrelated. The white genes have no relationship.

Mining of the genome for gene clusters involved in the biosynthesis of secondary metabolites has been performed by using the online antiSMASH tool 7.1.0 (https://antismash.secondarymetabolites.org/#!/about, accessed on 12 December 2024). In total, 38 regions were predicted, covering 23 NRPS and NRPS-likes, 12 T1PKS, 4 terpenes, 1 indole, 1 betalactone, 1 NRP-metallophore, and 1 T3PKS (Supplementary S1: Table S3). Based on antiSMASH prediction, we have found that *P. chrysogenum* INA 01369 has the potential to produce secalonic acids. One predicted cluster has 31% similarity to the known cluster of secalonic acid biosynthesis from *Claviceps purpurea* (accession: BGC0001886) (Figure 4) [26,27].

Three genes similar to known secalonic acid synthesis genes have been found in the cluster: polyketide synthase (identity: 50%), hypothetical tetrahydroxynaphthalene reductase (identity: 71%), and hypothetical AFLT efflux pump (aflatoxin efflux pump, identity: 50%). Two uncharacterized proteins with an identity of 54% and 59% have also been found. The rest of the predicted genes have been tested using BLASTP. Their products have turned out to be hypothetical and uncharacterized proteins.

### 2.4. Structure Elucidation of Compounds ***1*** and ***2***

**Figure 5:**
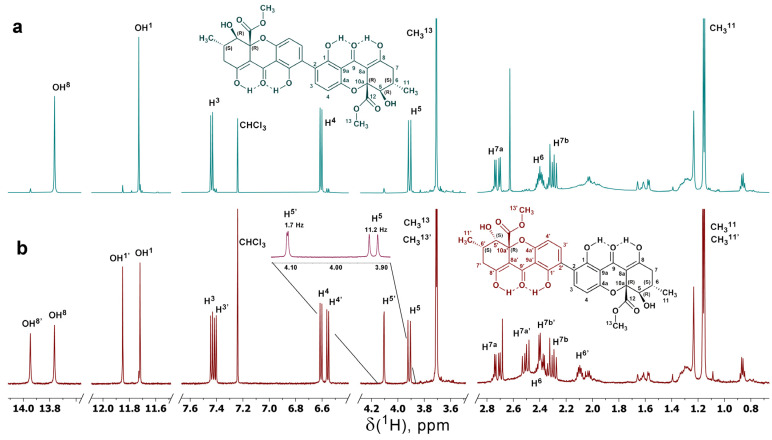
NMR ^1^H spectra of compounds **1** (**a**) and **2** (**b**) in CDCl_3_ recorded at 600 MHz frequency and 25 °C. Signal assignments are indicated for both compounds. In the spectrum of compound **2**, the H5 resonance region is expanded to highlight the key configurational difference between the two monomeric fragments of the molecule.

The structures of compounds **1** and **2** have been determined through detailed analysis of NMR and mass spectrometry (MS) data (Supplementary S2: Figure S3). The molecular mass of each compound, determined by MS, is 638 Da. In the ¹H NMR spectrum of compound **1**, signals corresponding to two methyl groups, four aliphatic resonances, two aromatic protons, and two peaks indicative of protons involved in strong hydrogen bonding were observed (Figure 5a). In contrast, the ¹H NMR spectrum of compound **2** shows a doubled set of similar signals (Figure 5b).

Resonance assignments have been achieved through the analysis of 2D NMR spectra, including DQF-COSY, TOCSY, ROESY, ¹³C-¹H HSQC, and ¹³C-¹H HMBC (Supplementary S2: Figures S4–S11), identifying two isoforms of secalonic acid, each with a dimeric tetrahydroxanthone structure. The ^1^H and ^13^C chemical shifts of compound **1** closely match published values for secalonic acid D [28,29], which is composed of two hemisecalonic E units linked via a C2–C2′ bond.

**Figure 6:**
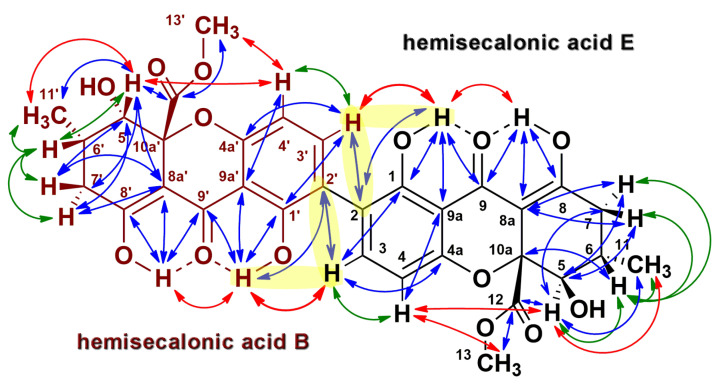
Correlations between the ¹H and ¹³C nuclei of secalonic acid F (ergochrome BE, compound **2**) as observed in the DQF-COSY (green arrows), ROESY (red arrows), and ¹³C-¹H HMBC (blue arrows) 2D NMR spectra. The correlations between the two monomeric subunits are highlighted in yellow.

In the ¹H NMR spectrum of compound **2**, one set of signals matches those of compound **1**, while the second set displays distinct variations. This observation suggests an asymmetric structure comprising a hemisecalonic acid E subunit linked to a hemisecalonic acid B subunit (Figure 6). It is important to observe that compound **2** is not a mixture of separate substances, as evidenced by correlations between the distinct monomeric subunits observed in the ROESY and ¹³C-¹H HMBC spectra (Figure 6, Supplementary S2: Figures S12 and S13).

The identification of monomeric subunits in compound **2**, specifically hemisecalonic acids E and B, was based on detailed NMR spectral analysis. These two isomers differ in the configuration of the asymmetric C5 carbon atom (Supplementary S2: Figure S14), resulting in variations in spin–spin coupling constants and nuclear Overhauser effects (NOEs) (Supplementary S2: Table S4). In hemisecalonic acid E, protons H5 and H6 are trans-oriented, resulting in the high value of the coupling constant, ^3^JH_5,H6_ = 11.2 Hz. In contrast, the gauche orientation of these protons in hemisecalonic acid B produces a much lower coupling constant of 1.7 Hz.

Furthermore, the C7 and C8a atoms in the hemisecalonic acid B fragment are trans to H5, whereas, in hemisecalonic acid E, they adopt a skewed conformation. This structural difference leads to prominent cross-peaks between H5 and C7/C8a in the ^13^C-^1^H HMBC spectrum of hemisecalonic acid B. In contrast, these correlations are either absent or significantly weaker for hemisecalonic acid E due to lower values of the corresponding coupling constants ^3^J_H5,C8a_ and ^3^J_H5,C7_.

Additionally, in hemisecalonic acid E, the H5 and C12 atoms are trans-oriented, whereas they are gauche in the B form. Consequently, the cross-peak between these resonances in the ^13^C-^1^H HMBC spectrum is significantly more intense for hemisecalonic acid E. Collectively, these data allow the unambiguous characterization of the configurations of the diastereomers E and B. An unsymmetrical tetrahydroxanthone dimer comprising two subunits, E and B, has been previously isolated from the fungus *Aspergillus aculeatus* and named secalonic acid F [29].

**Figure 7:**
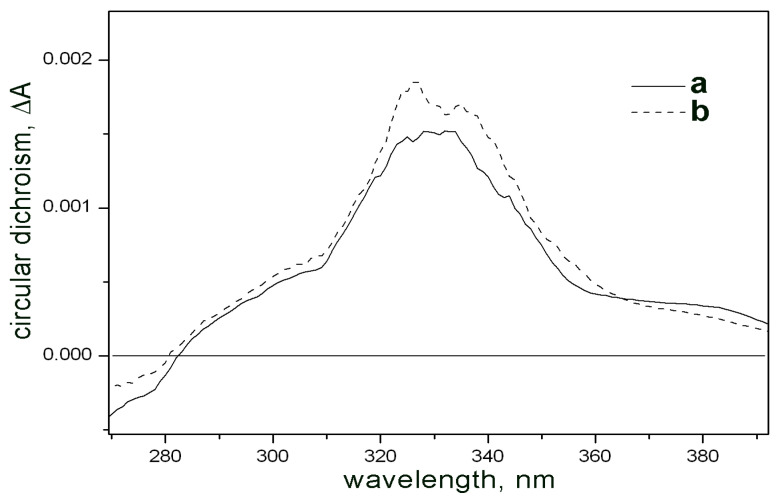
CD spectra of samples 1 (a) and 2 (b) in chloroform.

It is noteworthy that, based on NMR data, hemisecalonic acid E (10aR, 5R, 6S) is indistinguishable from its enantiomer, hemisecalonic acid A (10aS, 5S, 6R). Similarly, form B (10aR, 5S, 6S) is also enantiomeric to (10aS, 5R, 6R). To determine the absolute configurations of the studied compounds, circular dichroism (CD) spectra of both compounds **1** and **2** have been measured (Figure 7). Comparison of the CD spectra with those of the previously described blennolide isomers corresponding to the discussed hemisecalonic acids [29,30] corroborated the proposed structural assignments.

**Figure 8:**
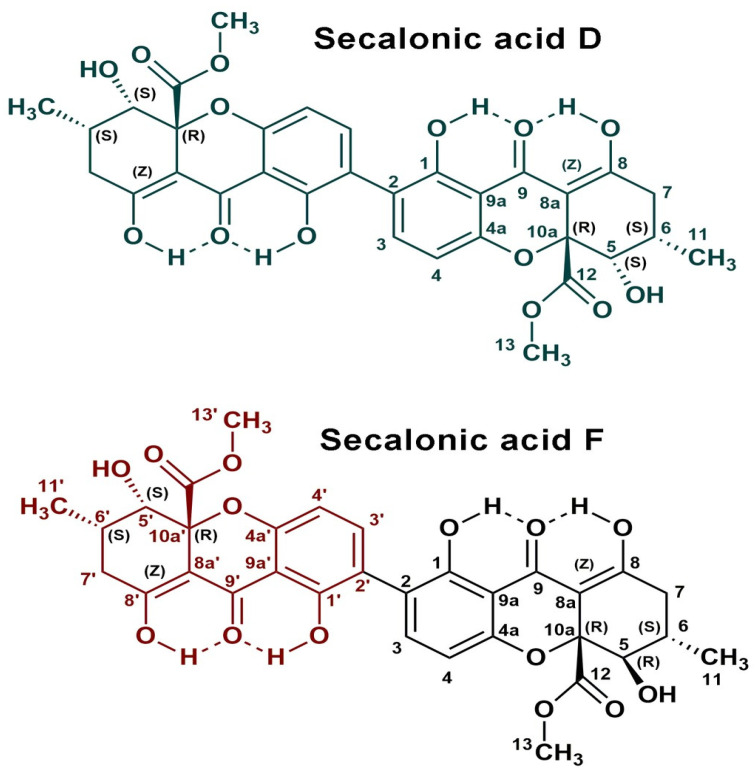
Structure of Secalonic acids D and F.

Table S4 (Supplementary S2) summarizes the NMR spectral parameters for hemisecalonic acids E and B measured in this study, alongside the previously published data for comparison. The chemical structures of compounds **1** and **2** are shown in Figure 8.

### 2.5. Antimicrobial Activities of Secalonic Acids

All of the isolated compounds have been evaluated for their antibacterial activities. Secalonic acid D exhibited a direct antibacterial effect at MIC of 4–16 μg/mL against three tested Gram-negative bacteria, including *E. coli* and *P. aeruginosa*, comparable to the conventional antibiotic ampicillin. Secalonic acid F also displayed strong antibacterial activity against MRSA *S. aureus* INA 00985, with an MIC value of 2 μg/mL. The most noteworthy result is that both secalonic acids F and D showed obvious activities against phytopathogenic bacteria *P. caratovorum* VKM-B1247 (Table 1) with MIC values of 4 and 16 μg/mL, respectively.

Secalonic acid D was not active against any of the *Aspergillus* species while secalonic acid F was active against *A. fumigatus* VKM F-37 and *A. niger* ATCC 16404, with MICs of 16 and 4 μg/mL, respectively.

Both of the tested compounds showed antifungal activity towards *C. albicans* ATCC 14053 and phytopathogenic strain *Fusarium solani* VKPM F 890. It has been found that secalonic acid F significantly inhibited the growth of all tested microorganisms.

## 3. Discussion

Extremophiles, including psychrophiles, have garnered attention for their potential applications in producing antibiotics, drugs, enzymes, and other biotechnological products [4,6,8,23,24,25]. A successful tendency to screen filamentous fungi in cold conditions for the creation of new bioactive compounds has been shown. To thrive in their harsh, climate-extreme settings, the cold-adapted fungi have developed a vast repertory of distinct secondary metabolites, both known and unknown. Numerous psychrophilic and psychrotolerant fungi demonstrate cytotoxic, antifungal, and antibacterial properties [2,4,6,23,24,25,31,32,33,34].

The fungi from the *Penicillium* genus have the potential to produce a large quantity of secondary metabolites, more than half of which have antimicrobial potential for human health and agriculture [1,20,21,22,35,36,37,38,39,40,41]. In particular, numerous compounds that include various classes and structures have been identified by both traditional and modern analytical methods from *P. chrysogenum* [5,7,24,25,31,32,33,34,42,43].

During our ongoing search for bioactive compounds, the psychrotolerant fungus *P. chrysogenum* INA 01369 from Northbrook Island was investigated. The present study reports the production of secalonic acids, secalonic acids D and F by strain INA 01369, and their antimicrobial and antifungal properties towards phytopathogenic and drug-resistant bacteria and fungi. Also, by mining the genome for gene clusters involved in the biosynthesis of secondary metabolites, we have predicted the gene cluster responsible for the biosynthesis of secalonic acids.

Taking into account the fact that the behavior and metabolism of microorganisms are affected differently by culture conditions, an analysis of different variables in these processes might amplify the ability of *P. chrysogenum* INA 01369 to produce secalonic acids in different conditions. The strain presents optimum growth at 20 °C and shows the ability to produce secalonic acids. The maximum number of secalonic acids was achieved in mycelium after 14 days of incubation at 20 °C in the stationary condition. The effect of temperature to repress or induce the secondary metabolism of filamentous fungi was previously reported by Keller et al. [34]. Furthermore, Pacios-Michelena et al. reported, in the case of the *P. chrysogenum* R1 strain, that the antifungal activity of cell-free extracts was significantly influenced by fermentation temperature [34,38].

In the study, we also reported the draft genome of a *P. chrysogenum* INA 01369. Phylogenetic analysis demonstrated that INA 01369 is a novel strain of *P. chrysogenum* species. In total, 37,547 protein-coding sequences and 10,500 mRNAs were predicted in the final official gene set of genSAS annotation. Augustus detected 37,457 protein-coding sequences and 10,500 mRNAs. Based on Pfam domain characterization, 8897 genes belonged to 3441 protein families and the GC content was 48.84%. The RepeatMasker program identified 7028 repeating regions. DIAMOD detected 10,083 proteins. tRNAscan-SE found 214 tRNAs. The genome size and GC content of this strain were similar to other environmental strains of *P. chrysogenum* [19,27,33,42].

The genome data and our bioinformatic analysis first revealed a large number of secondary metabolite clusters with no assigned compounds. In 2021, Matsuda’s team expressed the biosynthetic genes *Aacu* in *Aspergillus oryzae* to produce secalonic acids heterologously. The biosynthesis pathway of secalonic acids has been postulated based on the authors’ earlier research on the characterization (Nsr gene cluster) of the biosynthesis pathway [42]. In our genome mining study, three genes that resemble known genes involved in the synthesis of secalonic acid were identified in the cluster: hypothetical tetrahydroxynaphthalene reductase (identity: 71%), polyketide synthase (identity: 50%), and hypothetical AFLT efflux pump (identity: 50%). Two unidentified proteins with identities of 54% and 59% were also discovered. Strain INA 01369 has a large number of secondary metabolite clusters without corresponding compounds, according to the genomic data and our bioinformatic study.

Based on the chromatographic separation compounds and their characteristic absorption, two fractions have been obtained and further investigated. Both compounds I and II produced by the strain showed secalonic acids. However, secalonic acid F was obtained from the mycelium extract, while secalonic acid D was found in the extract from the culture liquid.

Secalonic acid families, usually referred to as xanthone dimers, are a class of bixanthones that are frequently extracted from lichen, plants, and fungi. Due to their biological activity, these molecules could have a potential range of applications in pharmaceutical fields [44,45,46,47,48,49,50,51,52,53]. Following their initial isolation in 1960, secalonic acids were discovered to possess intriguing bioactivities. By downregulating c-Myc, secalonic acid D exhibits strong cytotoxicity to HL60/K562 cells, while its diastereomer, secalonic acid B, possesses anticancer activity. Secalonic acid A, the enantiomer of D, exhibits anticancer qualities and lessens the toxicity of colchicine in rat cortical neurons [49,50,51]. Different forms of secalonic acid are generally reported to be produced by fungi from the genera *Aspergillus*, *Clohesyomyces*, *Cryptosporiopsis*, and *Paecilomyces*. Secalonic acid A was reported to be produced by the endophytic fungus *Diaporthe searlei* and had an antibacterial effect on multidrug-resistant bacterial strains, *S. aureus* 931 and *E. coli* 6720, and *Klebsiella pneumoniae* 815. Consequently, this molecule has shown MBCs of 4.7, 18.75, 37.5, and 75 μg/mL [44]. Furthermore, *Setophoma terrestris* obtained from the leaves litter in the mangrove ecosystem produced secalonic acid A and secalonic acid G with antimicrobial activity against *S. aureus* with MIC values of 75 μg/mL and 39 μg/mL, respectively [53].

Subsequently, findings of various species of *Penicillium* have been characterized by their ability to produce different secalonic acids—A [50,53], B [50], C [50], D [43,54,55,56], F, J–M [57]. Moreover, *Penicillium* spp. of section *Chrysogena* produces secalonic acid compounds with antimicrobial activity [50,53]. Secalonic acid D is the best-known and most commonly isolated compound among secalonic acids with antibacterial and cytotoxic properties [43,52,55,56,57]. Secalonic acid D isolated from *Penicillium* sp. SCSGAF 0023 and its epimer secalonic acid were able to inhibit biofilm formation in *S. aureus* by >90% at a concentration of 6.25 μg/mL without interfering with cell growth. Secalonic acid D showed the eradication of pre-developed biofilm and also formed sparse biofilm. Secalonic acid D exhibited a synergistic antibacterial and antibiofilm effect against *S. aureus*, with ampicillin, vancomycin, and chloramphenicol; the MIC was 2.5 μg/mL without secalonic acid D and 1 μg/mL with secalonic acid D [34]. Secalonic acids D and F from *P. chrysogenum* WX6 possess strong antimicrobial activity against *S. aureus* (ATCC12600), *Streptococcus mutans* (ATCC25175), *Pseudomonas fluorescens* (ATCC13525), *Moraxella catarrhalis* (ATCC25238), and *B. subtills* (ATCC6633), in comparison with secalonic acid A, which only exhibited activity towards *Pseudomonas fluorescens* (ATCC13525) [34]. In our study, it has been found that secalonic acid F also displayed strong antibacterial activity against MRSA *S. aureus* INA 00985, with an MIC value of 2 μg/mL. The most noteworthy result is that secalonic acid F showed obvious activities against the phytopathogenic bacteria *P. caratovorum* VKM-B1247 with an MIC value of 4 μg/mL. It was active also towards *A. fumigatus* VKM F-37 and *A. niger* ATCC 16404, with MICs of 16 and 4 μg/mL, respectively.

Environmental degradation and drug resistance are being caused by the overuse of synthetic fungicides for crop protection. Nonetheless, certain microbes have the ability to regulate infections. Chemical bactericides are not recommended for the control of soft rot bacteria and fungi due to their non-persistence and harmful side effects, as well as the fact that bacterial populations become more resistant [58,59]. All things considered, bioactive substances from the *Penicillium* genus hold the potential for transforming farming methods for the sustainable management of plant diseases. Thus, *P. brevicompactum* HE19ct isolated from *Piptatherium coerulescens* leaves showed an interesting wide spectrum of antimicrobial activities allowing the inhibition of Gram-positive bacteria, yeasts, and filamentous fungi *F. oxysporium* CTLM12 at 86.8 ± 2.0%. Contrary to results in a PDA-microplate, PDA-extract reduced its inhibition percentages (<30%) and spectrum against *Fusarium* strains, which only had antifungal activity against CTLM12 [59]. *P. chrysogenum* strain R1 has significant potential for controlling *F. oxysporum* by producing several secondary metabolites, which shows great promise for developing biological control processes [58]. Secalonic acid F has been previously derived from *P. chrysogenus* species [50,57]; however, little is known about its antimicrobial activity against phytopathogen bacteria *Pectobacterium caratovorum*, the causal agent of bacterial soft rot in vegetables [59]. Thus, secalonic acid F can be used for the biocontrol of phytopathogens. In our study, we have demonstrated, for the first time, the antimicrobial activity of the secalonic acid F against the phytopathogenic bacteria *Pectobacterium caratovorum,* which is a destructive soft rot disease to many economically important vegetables.

## 4. Materials and Methods

### 4.1. Strain of Penicillium chrysogenum INA 01369

The object of the present study was the psychrotolerant strain INA 01369 of the *Penicillium* sp. section *Chrysogena*. The strain was isolated in 2019 from the soil of seabird colonies (90 m above sea level) of Northbrook Island (Arkhangelsk region, Primorsky district, Franz Josef Land archipelago). The strain was cultivated in 2% malt extract agar (MEA: malt extract 20 g; bacteriological agar 15 g; deionized water 1000 mL) at 15 °C. The strain has been deposited into the Gause collection of antibiotic producers (the Gause Institute of New Antibiotics, Russia) with the number INA 13460 and the Russian National Collection of Industrial Microorganisms with the number VKPM F-1843. The strain has been maintained in the biobank by freezing at −80 °C using glycerol 10% and water, respectively. The fungal morphology was studied using a light microscope (LM) and scanning electron microscope (SEM). LM observations were performed on a Leica DM2500 microscope equipped with a DFC 495 camera. SEM observations were performed using JSM-6380LA (JEOL, Tokyo, Japan) and Quattro S (Thermo Scientific, Brno, Czech Republic) microscopes. Images were acquired and elaborated using MicroCapture (version 1.0) software.

### 4.2. DNA Isolation, Library Preparation, and Nanopore Sequencing

The genomic DNA of *Penicillium chrysogenum* INA 01369 was extracted from fungus culture cell pellets using DU. We used the cells, tissues, and blood DNA isolation kit (Biolabmix, Novosibirsk, Russia) in accordance with the manufacturer’s instructions. The integrity of the isolated DNA was checked using gel electrophoresis in a 1% agarose gel stained with ethidium bromide. The concentration of isolated DNA was assessed using a Nanodrop spectrophotometer (Nanodrop Technologies, Wilmington, NC, USA) and a Qubit 4 fluorimeter with a Qubit dsDNA HS assay kit (Thermo Fisher Scientific, Waltham, MA, USA). The DNA sample was adjusted to 400 ng in a 12 μL volume for the preparation of a nanopore sequencing library.

The ONT library (Oxford Nanopore Technologies, Oxford Science Park, Oxford, UK) was prepared using a Native Barcoding kit (SQK-NBD114-24; ONT, Oxford, UK), and the NEBNext companion module was used for ONT during DNA end repair and ligation (New England Biolabs, MA, USA). Long fragment library enrichment was performed using a long fragment buffer (LFB) in accordance with the manufacturer’s protocol. Sequencing was performed for 72 h on a MinION using R10.4.1 flow cells (FLO-MIN114; ONT, Oxford, UK) and MinKNOW v.22.10.7 software.

### 4.3. Phylogenetic Analysis and Genome Annotation

For the taxonomic identification of strain INA 01369, phylogenetic analysis was carried out using the maximum likelihood (ML). Several phylogenetic trees were constructed at different taxonomic levels to achieve a more precise determination of the taxonomic positions of the strain under study in the *Penicillium* genus. The nucleotide sequences for these constructs were obtained from the current revision of the *Penicillium* genus [1] and the NCBI database (Supplementary S1: Tables S1 and S2). For ML analyses, IQ-TREE 1.2.2 with the best-fitted model option was used. Bootstrapping was performed using the standard nonparametric bootstrap algorithm with the number of replicates set to 1000.

The de novo genome assembly of the strain INA 01369 of *P. chrysogenum* was generated by Nanopore sequencing data using Flye v. 2.9.3-b1797 with default parameters (https://www.nature.com/articles/s41587-019-0072-8, accessed 12 December 2024). Adapters removing and filtering short length (<200) and low quality (<8) reads were performed in fastp v. 0.23.4 (https://academic.oup.com/bioinformatics/article/34/17/i884/5093234, accessed 12 December 2024). After filtering, 99% of reads were retained. The read quality was assessed using NanoPlot v. 1.42.0 (https://www.ncbi.nlm.nih.gov/pmc/articles/PMC6061794/ accessed 12 December 2024). The genome was assembled using Flye v. 2.9.3-b1797 (https://www.nature.com/articles/s41587-019-0072-8 accessed 12 December 2024) with default parameters. Polishing of the draft assembly was performed using Racon v. 1.5.0 (https://www.ncbi.nlm.nih.gov/pmc/articles/PMC5411768/ accessed 12 December 2024) and Medaka v. 1.11.2 (https://github.com/nanoporetech/medaka accessed 12 December 2024). The assembly was corrected by removing contigs with short lengths (>500 nt) and low coverage (>10 nt). Scaffolding was performed with RagTag v2.1.0 (https://genomebiology.biomedcentral.com/articles/10.1186/s13059-022-02823-7, accessed 12 December 2024). The final assembly contains 5 scaffolds and 84 contigs. The total length of the assembly—33,708,980 nt, N50—7,242,977, L50—3.

Annotation was performed with the online tool GenSAS 6.0 (https://www.gensas.org/gensas, accessed 12 December 2024) using RepeatMasker 4.1.1 (https://www.repeatmasker.org/, accessed 12 December 2024), EvidenceModeler 1.1.1 (https://github.com/EVidenceModeler/EVidenceModeler/wiki, accessed 12 December 2024), Augustus 3.4.0 (https://github.com/Gaius-Augustus/Augustus, accessed 12 December 2024), DIAMOND 2.0.6 (https://github.com/bbuchfink/diamond, accessed 12 December 2024), BLAST+ 2.12.0 (https://blast.ncbi.nlm.nih.gov/doc/blast-help/downloadblastdata.html#downloadblastdata, accessed 12 December 2024), getorf 6.6.0 (https://emboss.sourceforge.net/apps/cvs/emboss/apps/getorf.html, accessed 12 December 2024), tRNAscan-SE 2.0.7 (https://lowelab.ucsc.edu/tRNAscan-SE/, accessed 12 December 2024) programs, and Pfam database 1.6 (http://pfam.xfam.org/, accessed 12 December 2024). Fungal secondary metabolite pathways were predicted using the online tool antiSMASH 7.1.0 (https://antismash.secondarymetabolites.org/#!/about, accessed 12 December 2024).

### 4.4. The Effect of Different Temperatures and Cultivation Conditions on Fungal Growth and Antimicrobial Activity

Culture growth was examined at different temperatures, namely, 5 °C, 10 °C, 15 °C, 25 °C, and 30 °C on Potato Dextrose agar (PDA, pH 7) and Chapek Dox agar (CZA, pH 7). To elucidate the psychrotolerant adaptation of the strain, we studied the fungal growth properties and antibiotic activity at several temperatures: 15 °C, 20 °C, and 25 °C on a Potato Dextrose Broth (PDB) medium according to the recommendations provided by Haasan et al. [60]. The producer strain was grown in 750 mL Erlenmeyer flasks with 350 mL medium for 14 days. We performed the stationary or the submerged method on an Innova 40R shaker–incubator (Eppendorf, New Brunswick, NJ, USA). Each experiment was carried out in triplicate.

### 4.5. Purification and Identification of the Secalonic Acid Derivates

#### 4.5.1. HPLC Analysis

The culture liquid (CL) was separated via filtration through membrane filters on a Seitz funnel under vacuum. Then, CL was extracted three times with ethyl acetate (EtOAc), methanol, or butanol at the ratio of 5:1. The obtained extracts were evaporated in a vacuum on a Rotavapor-RBüchi rotary evaporator (Büchi, Flawil, Switzerland) at 42 °C to dryness; the residue was dissolved in aqueous 50% ethanol, and alcoholic concentrates were obtained. The concentrates were analyzed and separated into active fractions using semi-preparative reverse-phase high-performance liquid chromatography (RP-HPLC) with an XBridge BEH C18 OBD Prep Column, 130Å, 5 µm, 10 mm × 250 mm (Waters, Milford, MA, USA) in a linear gradient of increasing concentration of acetonitrile as a mobile phase (eluent A, 0.1% trifluoroacetic acid, TFA, in MQ deionized water; eluent B, 80% acetonitrile with 0.1% aqueous TFA) at a flow rate of 2.0 mL/min. The separated substances were detected at a wavelength of 220 nm and collected for testing. The re-chromatography of the active fractions has been carried out using an analytical column, Luna 5 µm C18 250 × 4.6 mm (Phenomenex, Torrance, CA, USA) at a flow rate of 1.0 mL/min.

#### 4.5.2. Mass Spectrometry

Electron ionization mass spectra were recorded with a Finnigan MAT INCOS 50 spectrometer with direct injection of a sample into the ion source with an ionization energy of 70 eV and a controlling voltage of 1.75 kV.

#### 4.5.3. NMR Spectroscopy

NMR spectra were acquired on a Bruker AVANCE spectrometer operating at 600 MHz for ^1^H at 298 K, using CDCl_3_ and DMSO-d_6_ as the solvents at a concentration of approximately 8 mM. Assignments of ^1^H and ^13^C signals at natural abundance were obtained using a set of 2D experiments: DQF-COSY, TOCSY, ROESY, ^13^C-^1^H HSQC, and ^13^C-H HMBC. The mixing times for TOCSY and ROESY experiments have been set to 80 and 320 ms, respectively.

^1^H and ^13^C chemical shifts were referenced to TMS as an internal standard. Spectral processing was performed using NMRPipe [61] following a standard protocol that included Lorentz-to-Gauss window functions, forward–backward linear prediction, and polynomial baseline correction. Additionally, 2D spectra were analyzed with NMRFAM-Sparky [62] and 1D NMR spectra were processed and analyzed using Mnova software v. 12.0.0 (Mestrelab Research, Spain).

#### 4.5.4. Circular Dichroism Spectroscopy

Circular dichroism (CD) spectra were recorded on a Chirascan CD spectrometer (Applied Photophysics, Leathenhead, UK).

### 4.6. Antimicrobial Activity Assay

The initial spectrum of the antimicrobial action of the substances contained in the fractions has been determined via disk diffusion. The antimicrobial activity has been determined in the original CL, in the alcoholic extracts of CL, and in the mycelium extracts with sterile paper disks (St. Petersburg Pasteur Institute, St. Petersburg, Russia) soaked in extracts and dried under sterile conditions. Standard disks with Amphotericin B for fungi (40 μg, St. Petersburg Pasteur Institute, Russia) and Ampicillin for bacteria (20/10 μg, St. Petersburg Pasteur Institute, Russia) were used as controls.

The Minimal Inhibition Concentration (MIC) for bacteria and fungi was determined in a 96-well plate using the microdilution method in LB broth medium for bacteria and RPMI 1640 medium for fungi. The compounds and the positive control were dissolved in dimethyl sulfoxide (DMSO) in a two-fold serial dilution at concentrations ranging from 0.5 to 200 mg/mL. The MIC values of each individual compound were determined using the broth twofold microdilution method according to CLSI/NCCLS documents M27-A3, M38-A, and M38-A [63,64,65]. Ampicillin and amphotericin B (ranging from 0.0625 to 4 μg/mL) served as controls for bacteria and fungi, respectively. DMSO was used as a control for the carrier. Fungal and bacterial test-strains *Candida albicans* ATCC 14053 *Aspergillus niger* ATCC 16404, *Pseudomonas aeruginosa* ATCC 27853, *Escherichia coli* ATCC 25922, *Staphylococcus aureus* INA 00985 MRSA, and *Fusarium solani* VKPM F 890 were obtained from the collection of the Gause Institute of New Antibiotics (Moscow, Russia). *Aspergillus fumigatus* VKM F-37 and *P. caratovorum* VKM-B1247 have been provided by the All-Russia Collection of Microorganisms (VKM, Pushchino, Russia). Each experiment was carried out in triplicate.

## 5. Conclusions

In our work, we have studied the biological properties of a novel psychrotolerant strain of the fungus *P. chrysogenum* INA 01369 that has been isolated at the Franz Josef Land archipelago. The whole genome data and our bioinformatics analysis have revealed that this strain contains a large number of secondary metabolite clusters without assigned compounds. We have isolated antibiotics produced under optimal conditions for this psychrotolerant strain. Our chemical investigation indicates that INA 01369 produces secalonic acids D and F as the dominant antimicrobials. These metabolites exhibited a wide range of antimicrobial activities against the MRSA human pathogen, phytopathogenic bacteria, and fungi. Thus, the data obtained provide evidence for *P. chrysogenum* INA 01369 to be a potential natural source for antimicrobial drug discovery and biological control applications. On the other hand, the present study leaves open the question of the ecological significance of the production of secalonic acids by saprotrophic fungi in cold Arctic environments.

## Figures and Tables

**Table 1:** Antimicrobial activities of compounds **1**–**2** (MIC, μg/mL).

Strain	Secalonic Acid D	Secalonic Acid F	Ampicillin	Amphotericin B
*P. aeruginosa* ATCC 27853	16	8	4	nt
*E. coli* ATCC 25922	4	4	4	nt
*S. aureus* INA 00985 MRSA	8	2	>64	nt
*P. caratovorum* VKM-B1247	16	4	2	nt
*F. solani* VKPM F 890	8	8	nt	0.5
*A. fumigatus* VKM F-37	>64	16	nt	2
*C. albicans* ATCC 14053	8	4	nt	0.5
*A. niger* ATCC 16404	>32	4	nt	2

nt—non-tested.

## Data Availability

All sequence data are available in NCBI GenBank following the accession numbers in the manuscript.

## Supplementary Materials

The following supporting information can be downloaded at: https://www.mdpi.com/article/10.3390/antibiotics14010088/s1, Supplementary S1: Table S1. Sequences of fungal *Penicillium* species of *Penicillium* subgenus used in this study with their GenBank accession numbers; Table S2. Sequences of fungal *Penicillium* species of *Chrysogena* section (subgen. *Penicillium*) used in this study with their GenBank accession numbers; Figure S1. Growth of the strain INA 01369 in different media: (A) Chapek Dox medium and (B) PDA medium; Figure S2. HPLC profile of the separation of EtOAc extract from the producer mycelium (sample diluted in DMSO 1:5 *v*/*v*). Separation using a semi-preparative C18 column, absorption detection at 220 nm. Active fractions are labeled by release time (A). Subsequent rechromatography of the antimicrobial fractions, identified as secalonic acids D (B) and F (C) using an analytical C18 column; Table S3. Biosynthetic gene cluster analysis of the strain INA 01369 using ANTISMASH. Supplementary S2: Figure S3. Mass spectrum; Figures S4–S13. NMR spectra; Figure S14: Stereochemistry of hemisecalonic units; Table S4. Chemical shifts of secalonic acids D and F. References [28,29] are cited in the Supplementary Materials.
