# Supplementary material for: Antimicrobial Potential of Secalonic Acids from Arctic-Derived Penicillium chrysogenum INA 01369
Source: Antibiotics (Basel). 2025 Jan 14;14(1):88. doi: 10.3390/antibiotics14010088 (PMC11761870; doi:10.3390/antibiotics14010088)
Supplement: Supplementary file 1 [file antibiotics-14-00088-s001.zip › antibiotics-3415883-supplementary.pdf]

Supporting Information 1

for

Antimicrobial Potential of Secalonic Acids from Arctic-Derived *Penicillium chrysogenum*

Yulia A. Roshka[1], Natalia N. Markelova[1], Sofia D. Mashkova[1], Kseniya V. Malysheva[1], Marina L. Georgieva[1,2], Igor B. Levshin[1], Vladimir I. Polshakov[3], Alexander M. Arutyunian[4], Aleksey S. Vasilchenko[4] and Vera S. Sadykova[1*]

[1] Laboratory for Taxonomic Study and Collection of Cultures of Microorganisms, Gause Institute of New Antibiotics, st. Bolshaya Pirogovskaya, 11, 119021 Moscow, Russia; sadykova_09@mail.ru;
[2] Faculty of Biology, Lomonosov Moscow State University, 1-12 Leninskie Gory, 119234 Moscow, Russia; kokaeval@gmail.com; i-marina@yandex.ru
[3] Center for Magnetic Tomography & Spectroscopy, Faculty of Fundamental Medicine, Lomonosov Moscow State University, Leninskie Gory, GSP-1, Moscow, 119991, Russia vpolsha@fbm.msu.ru
[4] A.N. Belozersky Institute of Physico-Chemical Biology, Lomonosov Moscow State University, Leninskie Gory, GSP-1, Moscow, 119991, Russia; alarut@belozersky.msu.ru
[5] Laboratory of Antimicrobial Resistance, Institute of Environmental and Agricultural Biology (X-BIO), Tyumen State University, Tyumen, Russian Federation

Table of contents:

1	Table S1-S2	Sequences *Penicillium* species	2-3
2	Figures S1	Growth of the strain on agar media	4
3	Figure S2	HPLC profile	5
4	Table S3	Biosynthetic gene cluster analysis	6-7

Table S1. Sequences fungal *Penicillium* species of *Penicillium* subgenus used in this study with their GenBank accession numbers

	**Species**	**Isolate No.**	**GenBank accession No. (ITS)**
1	*Penicillium brevicompactum*	CBS 257.29^{T}	AY484912
2	*P. canescens*	CBS 300.48^{T}	AF033493
3	*P. chrysogenum*	CBS 306.48^{T}	AF033465
4	*P. expansum*	CBS 325.48^{T}	AY373912
5	*P. formosanum*	CBS 211.92^{T}	KC411696
6	*P. hirsutum*	CBS 135.41^{T}	AY373918
7	*P. lanosum*	CBS 106.11^{T}	DQ304540
8	*P. osmophilum*	CBS 462.72^{T}	EU427295
9	*P. paradoxum*	CBS 527.65^{T}	EF669707
10	*P. robsamsonii*	CBS 140573^{T}	KU904339
11	*P. roqueforti*	CBS 221.30^{T}	HQ442347
12	*P. sacculum*	CBS 231.61^{T}	KC411707
13	*P. turbatum*	CBS 383.48^{T}	AF034454
	Outgroup		
14	*Penicillium glabrum* (subgen. *Aspergilloides*, sect. *Aspergilloides*)	CBS 125543^{T}	GU981567

T - ex-type strains

Table S2. Sequences fungal *Penicillium* species of *Chrysogena* section (subgen. *Penicillium*) used in this study with their GenBank accession numbers

	Species	Isolate No.	GenBank accession No.		
			BenA	CaM	RPB2
	series *Aethiopica*				
1	*Penicillium lanosocoeruleum*	CBS 215.30	KU896817	JX996967	JX996723
	series *Chrysogena*				
2	*P. allii-sativi*	CBS 132074	JX996891	JX996232	JX996627
3	*P. chrysogenum*	CBS 306.48	JF909955	JX996273	JN121487
4	*P. confertum*	CBS 171.87	AY674373	JX996963	JX996708
5	*P. desertorum*	CBS 131543	JX996818	JX996937	JX996682
6	*P. dipodomyus*	CBS 110412	AY495991	JX996950	JF909932
7	*P. flavigenum*	CBS 419.89	AY495993	JX996281	JN406551
8	*P. halotolerans*	CBS 131537	JX996816	JX996935	JX996680
9	*P. mononematosum*	CBS 172.87	AY495997	JX996964	JX996709
10	*P. nalgiovense*	CBS 352.48	KU896811	JX996974	JX996719
11	*P. rubens*	CBS 129667	JF909949	JX996263	JX996658
12	*P. tardochrysogenum*	CBS 132200	JX996898	JX996239	JX996634
13	*P. vanluykii*	CBS 131539	JX996879	JX996220	JX996615
	series *Crustacea*				
14	*P. egyptiacum*	CBS 244.32	KU896810	JX996969	JN406598
15	*P. kewense*	CBS 344.61	KU896816	JX996973	JF417428
16	*P. sinaicum*	CBS 279.82	KU896818	JX996970	JN406587
	series *Goetziorum*				
17	*P. goetzii*	CBS 285.73	KU896815	JX996971	JX996716
	series *Persicina*				
18	*P. persicinum*	CBS 111235	JF909951	JX996954	JN406644
	Outgroup				
	P. hirsutum (sect. *Fasciculata*, ser. *Corymbifera*)	CBS 135.41	MN969384	KU896840	JN406629

T - ex-type strains

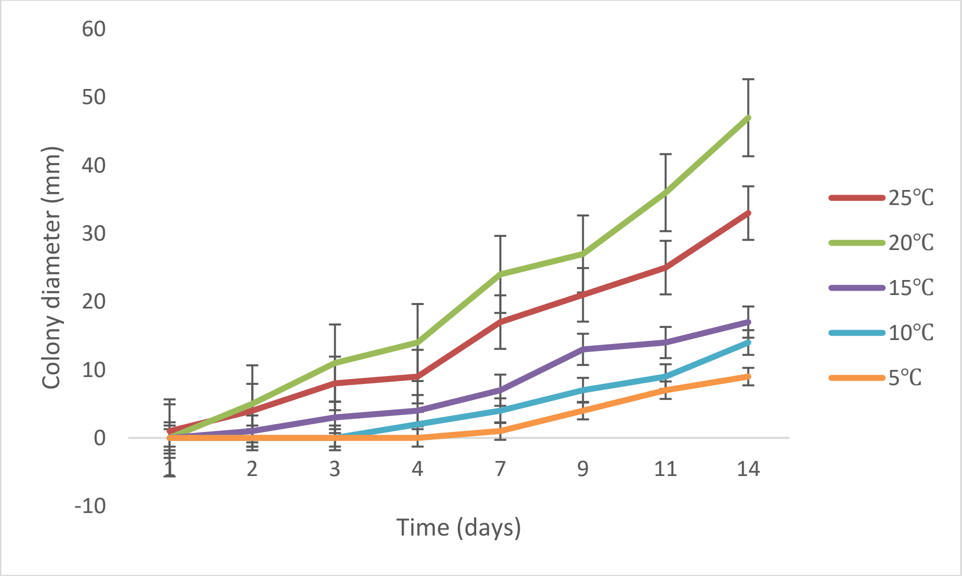

A

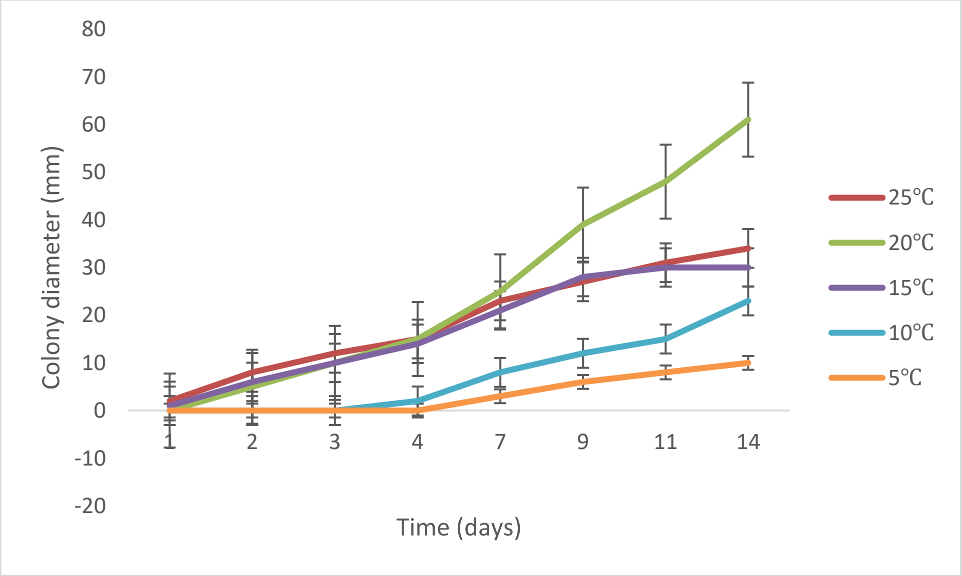

B

Figure S1. Growth of the strain INA 01369 at different media (A) – Chapek-Dox medium and (B) – PDA medium.

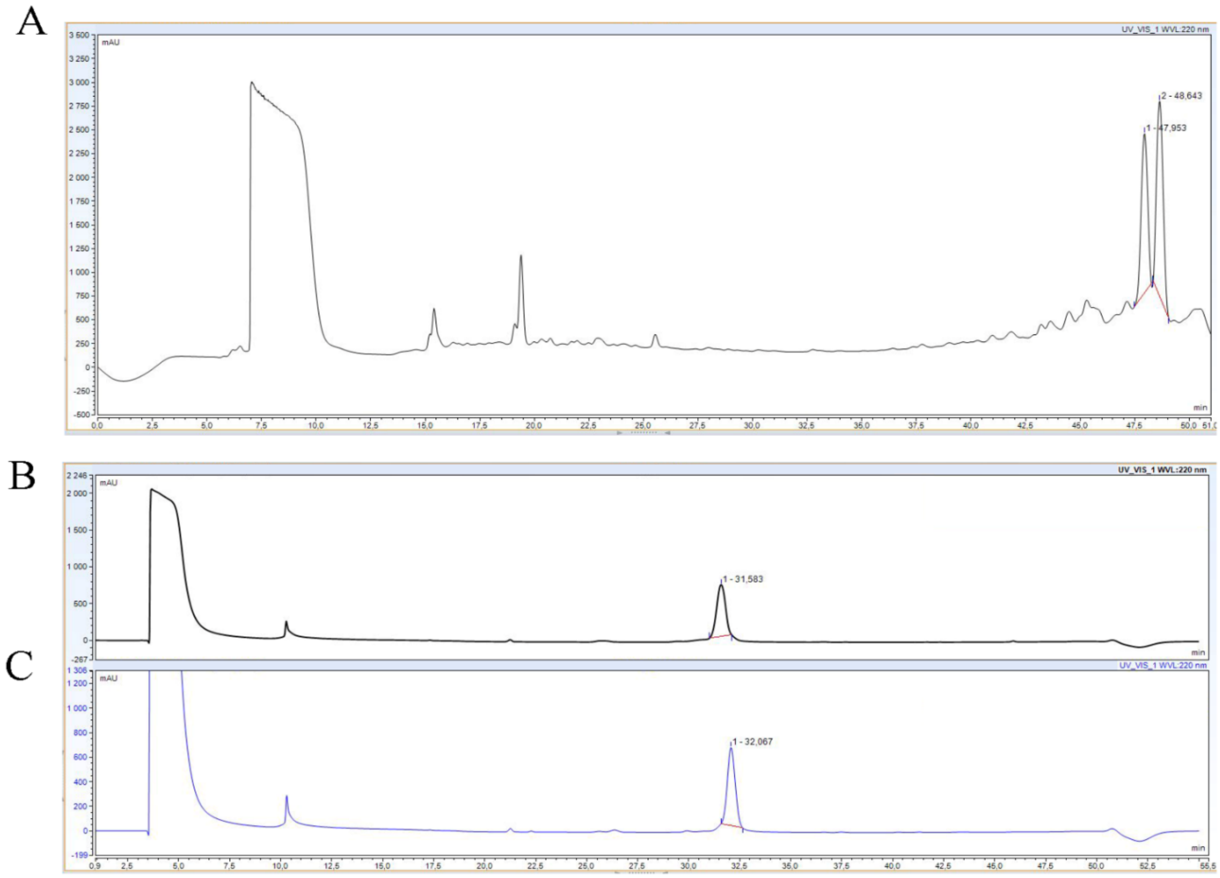

Figure S2. HPLC profile of the separation of EtOAc extract from the producer mycelium (sample diluted in DMSO 1:5 v/v). Separation using a semi-preparative C18 column, absorption detection at 220 nm. Active fractions are labeled by release time (A). Subsequent rechromatography of the antimicrobial fractions, identified as secalonic acid D (B) and F (C) using an analytical C18 column.

Table S3. Biosynthetic gene cluster analysis of the strain INA 01369 using ANTISMASH

Region	Type	From	To	Most similar known cluster	Similarity	%
Region 1.1	NRPS-like	135,856	198,881			
Region 2.1	T1PKS	824,930	888,888	cryptosporioptide B/cryptosporioptide A/cryptosporioptide C	Polyketide:Iterative type I polyketide	34%
Region 3.1	T1PKS	1,335	119,025	sorbicillin	Polyketide	71%
Region 3.2	NRPS	661,044	705,792			
Region 4.1	T1PKS,NRPS-like	517,568	583,209			
Region 5.1	NRPS-like	386,062	450,201	choline	NRP	100%
Region 10.1	NRPS	365,264	430,199	chrysogine	NRP	66%
Region 11.1	T1PKS	308,091	377,017			
Region 12.1	T1PKS,NRPS	88,470	169,609	metachelin C/metachelin A/metachelin A-CE/metachelin B/dimerumic acid 11-mannoside/dimerumic acid	NRP	50%
Region 13.1	NRPS-like	161,490	219,369			
Region 13.2	NRPS-like	279,482	341,911	azasperpyranone A/azasperpyranone B/azasperpyranone C/azasperpyranone D/azasperpyranone E/azasperpyranone F/azasperpyranone G/azasperpyranone H	Polyketide	12%
Region 16.1	NRPS	303,598	339,666			
Region 20.1	terpene	201,871	233,350	squalestatin S1	Terpene	60%
Region 24.1	NRPS-like,NRPS	109,204	231,006	FR901483	NRP	25%
Region 27.1	T1PKS	1	61,132	patulin	Polyketide:Iterative type I polyketide	40%
Region 30.1	betalactone	108,475	149,033			
Region 30.2	NRPS-like	224,516	269,329			
Region 31.1	T1PKS	47,027	117,350			

Region 31.2	terpene	164,782	196,154			
Region 32.1	betalactone	79,597	113,812			
Region 35.1	T1PKS	25,491	93,490			
Region 39.1	T3PKS	52,909	115,841			
Region 43.1	T1PKS	63,682	125,887			
Region 45.1	T1PKS	139,745	205,075			

Supporting Information 2

for

Table of contents:

1	Figure S3	Mass-spectrum	9
2	Figures S4-S13	NMR spectra	10-19
3	Figure S14	Stereochemistry of hemisecalonic units	20
4	Table S4	Chemical shifts of secalonic acids D and F	21

Figure S3. Mass-spectrum of the compound 1.

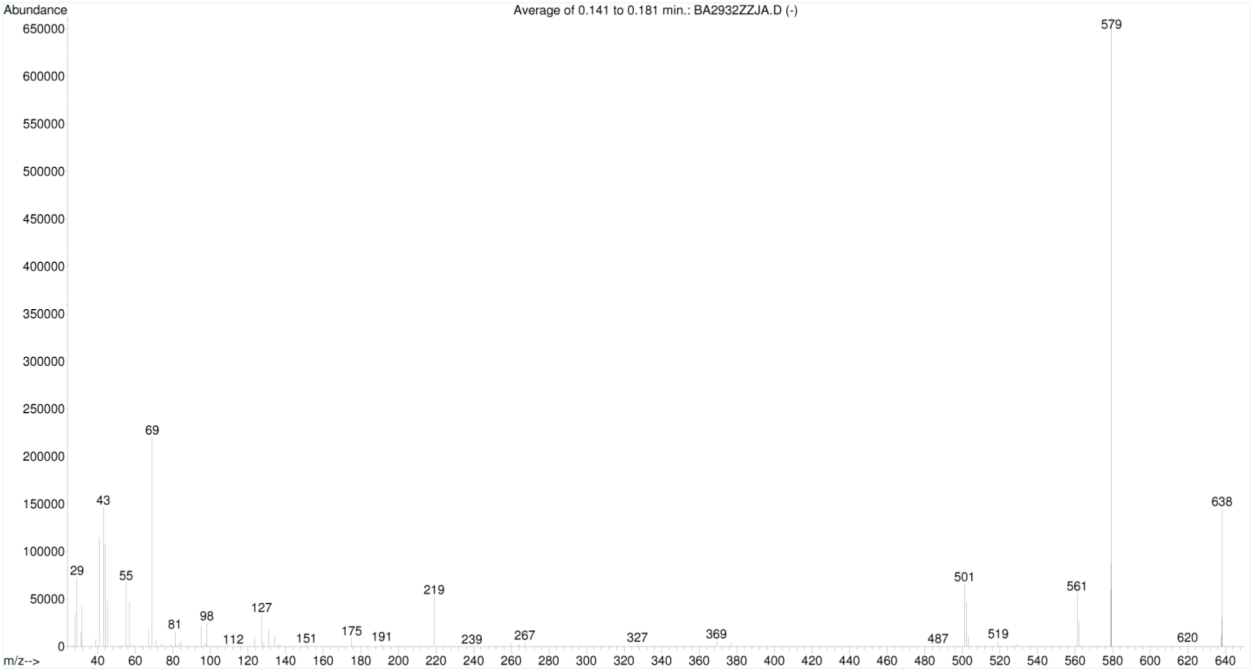

Figure S4. 2D DQF COSY spectrum of the compound compound **1** (secalonic acid D, composed of two identical hemisecalonic units E) recorded in $CDCl_3$ at 600 MHz and 298K.

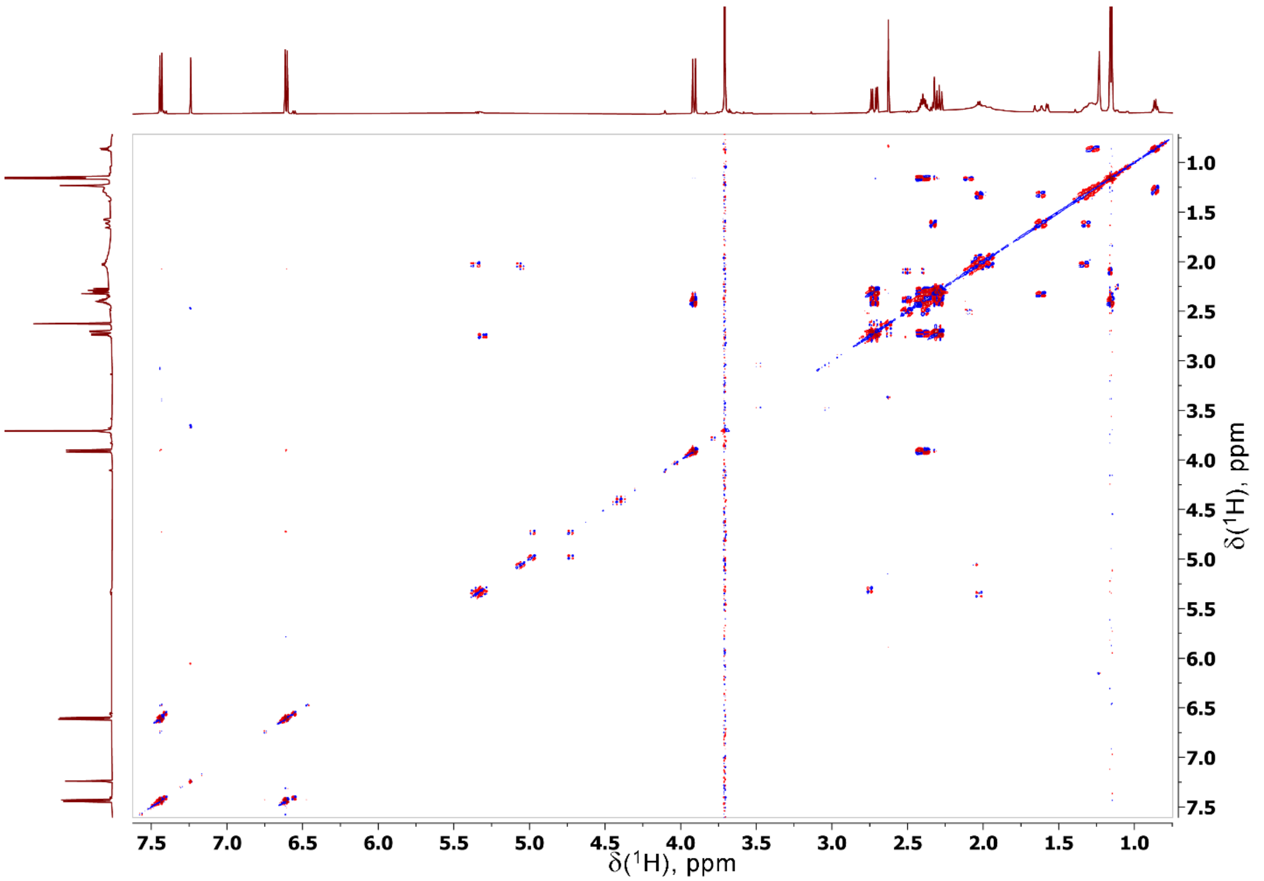

Figure S5. 2D ROESY spectrum (350 ms mixing time) of the compound **1** (secalonic acid D, composed of two identical hemisecalonic units E) recorded in $CDCl_3$ at 600 MHz and 298K.

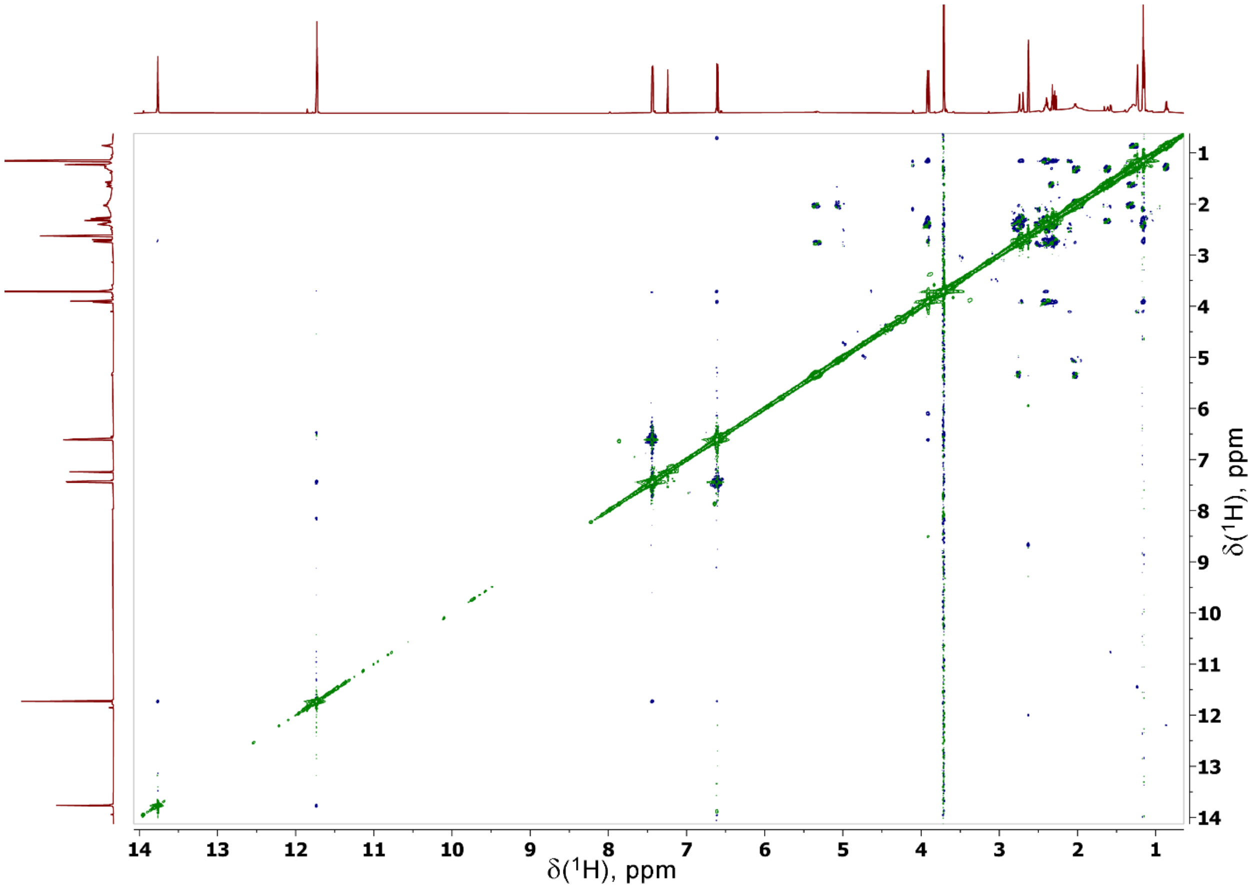

Figure S6. 2D ^{13}C-^{1}H HSQC spectrum of the compound **1** (secalonic acid D, composed of two identical hemisecalonic units E) recorded in $CDCl_3$ at 600 MHz and 298K.

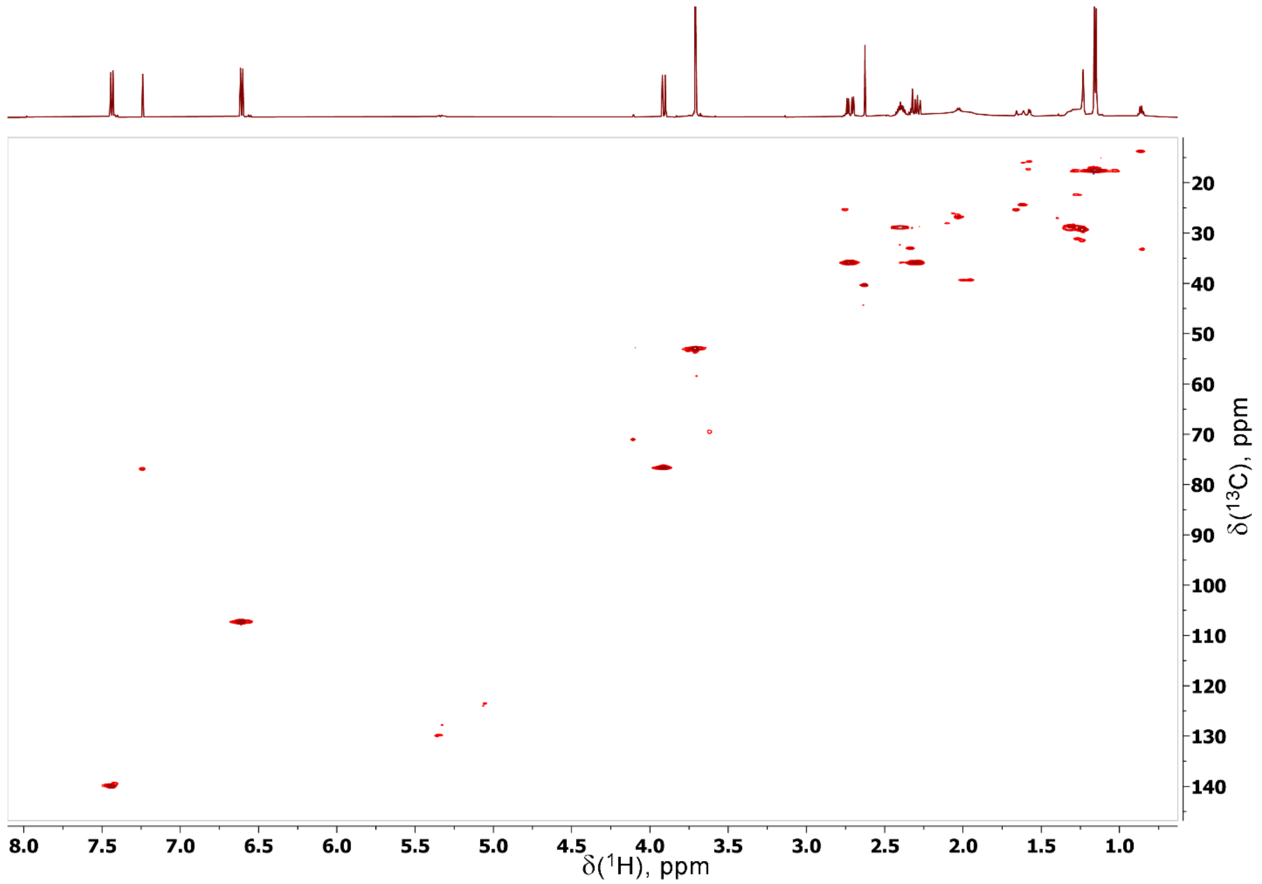

Figure S7. Overlay of the 2D ^{13}C-^{1}H HSQC (blue signals) and ^{13}C-^{1}H HMBC (red signals) spectra of the compound **1** (secalonic acid D, composed of two identical hemisecalonic units E) recorded in $CDCl_3$ at 600 MHz and 298K. Shown are resonance assignments.

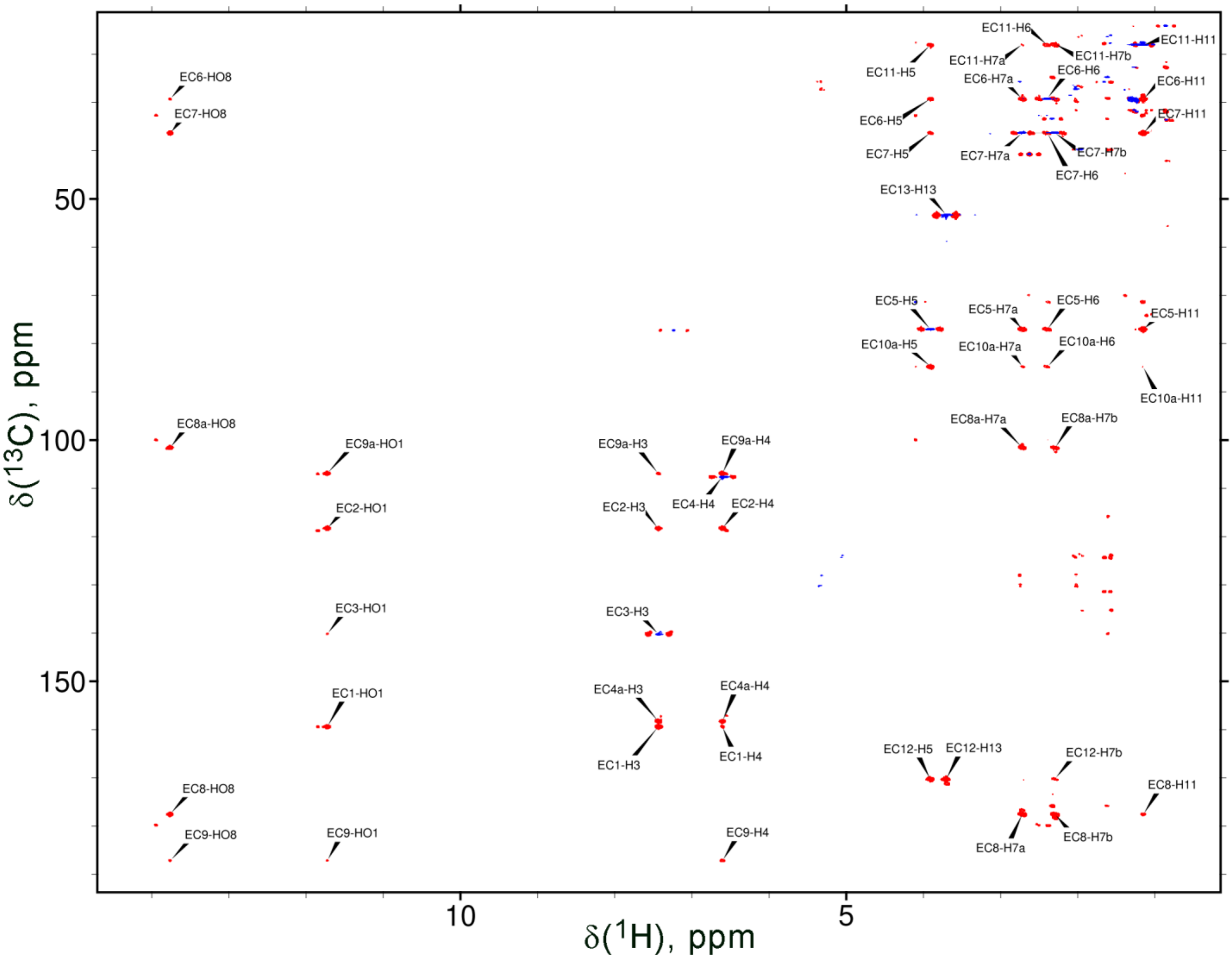

Figure S8. 2D DQF COSY spectrum of the compound **2** (secalonic acid F, composed of hemisecalonic units E and B) recorded in $CDCl_3$ at 600 MHz and 298K.

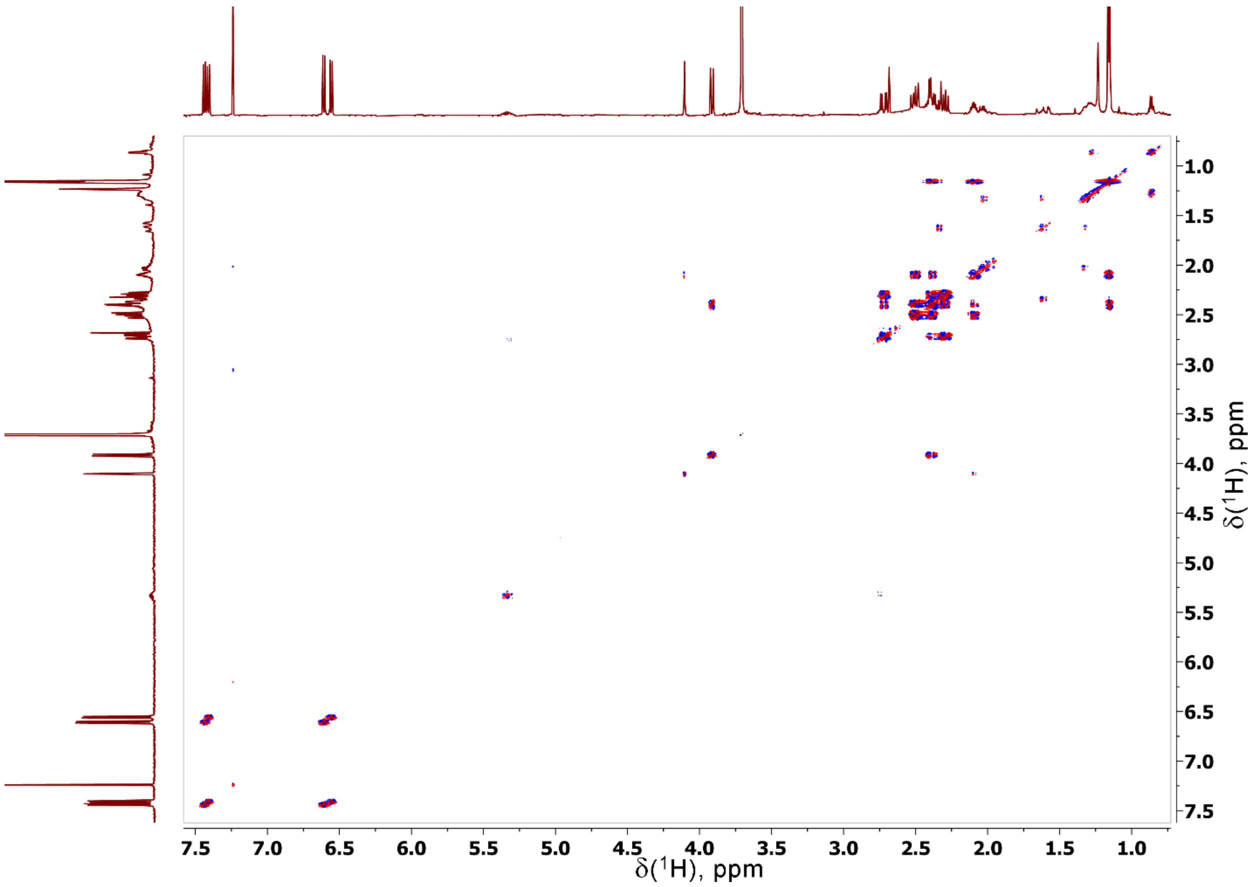

Figure S9. 2D ROESY spectrum of the compound **2** (secalonic acid F, composed of hemisecalonic units E and B) recorded in $CDCl_3$ at 600 MHz and 298K.

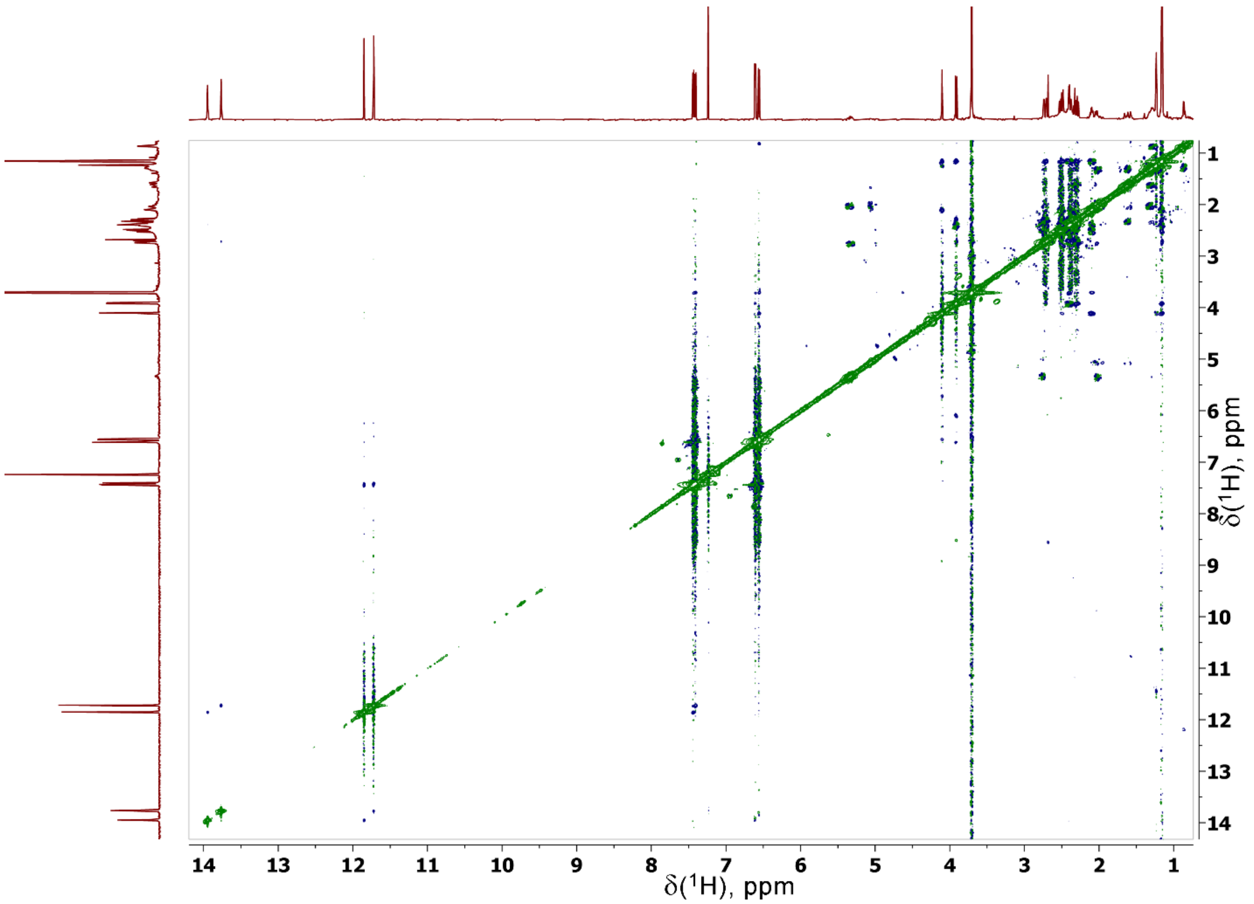

Figure S10. 2D ^{13}C-^{1}H HSQC spectrum of the compound **2** (secalonic acid F, composed of hemisecalonic units E and B) recorded in $CDCl_3$ at 600 MHz and 298K.

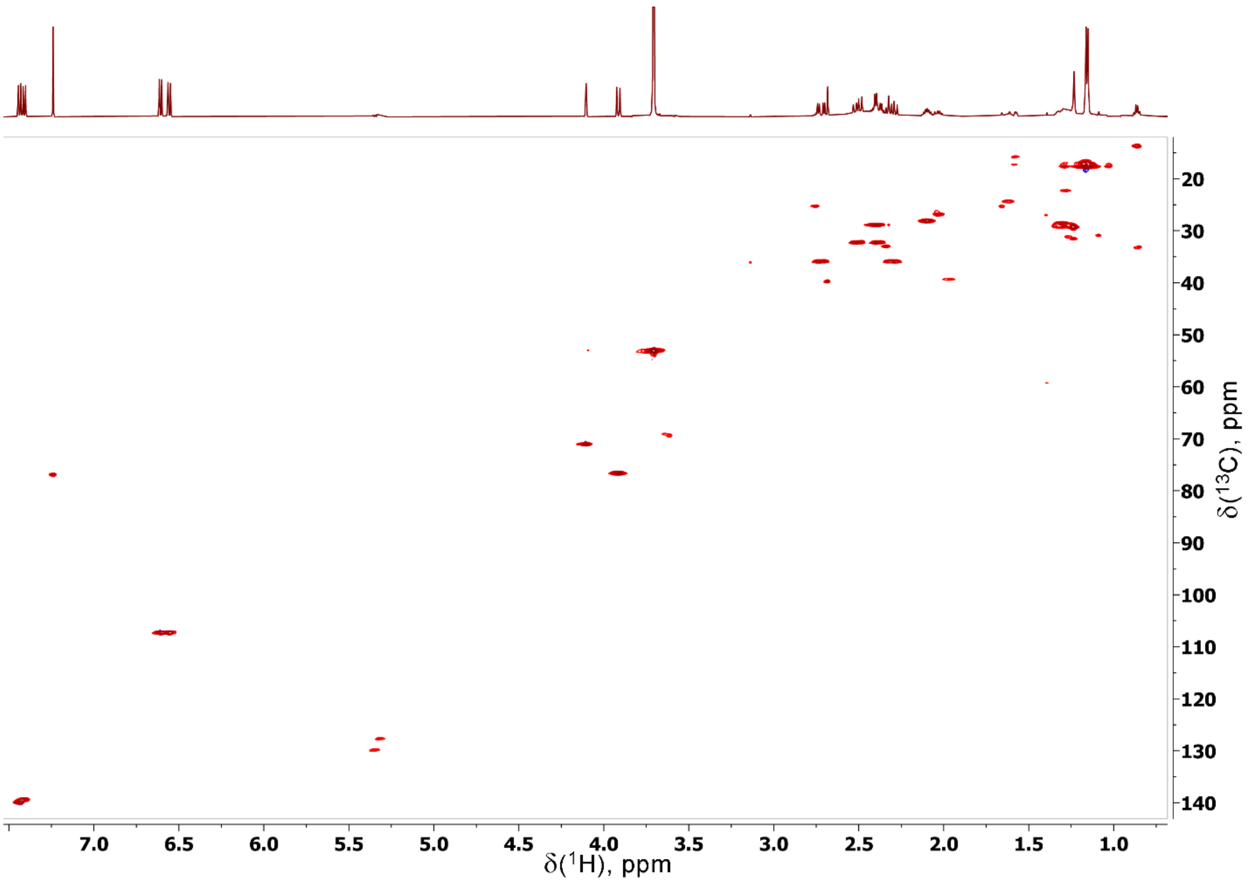

Figure S11. Overlay of the 2D ^{13}C-^{1}H HSQC (blue signals) and ^{13}C-^{1}H HMBC (red signals) spectra of the compound **2** (secalonic acid F, composed of hemisecalonic units E and B) recorded in $CDCl_3$ at 600 MHz and 298K. Shown are resonance assignments.

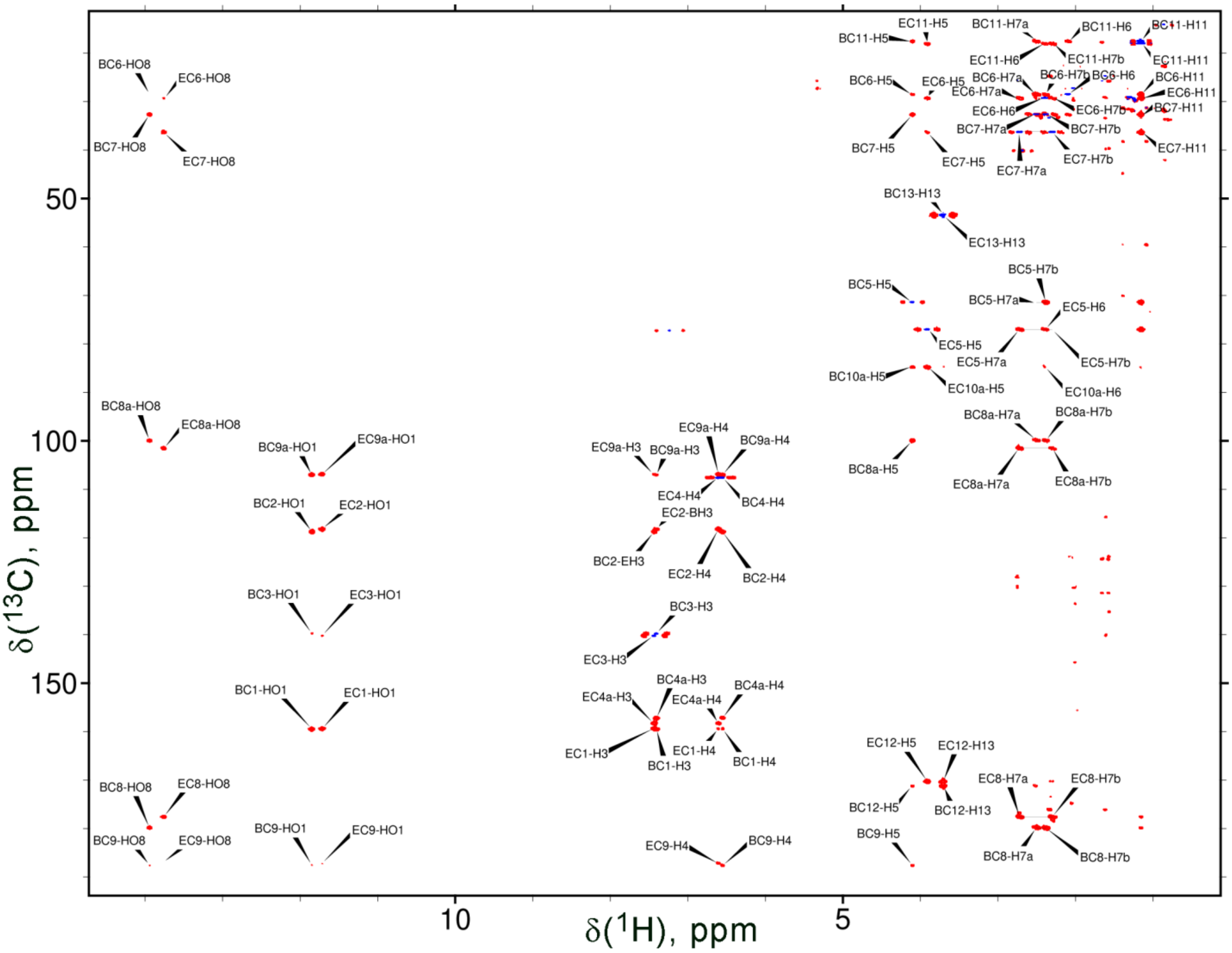

Figure S12. Fragment of the ^{13}C-^{1}H HMBC spectrum of the compound **2** (secalonic acid F, composed of hemisecalonic units E and B) recorded in $CDCl_3$ at 600 MHz and 298K. Correlations between C2 and H3 nuclei from different (E and B) hemisecalonic units are shown. For clarity, intraunit correlations between C2 and OH1 nuclei are also shown.

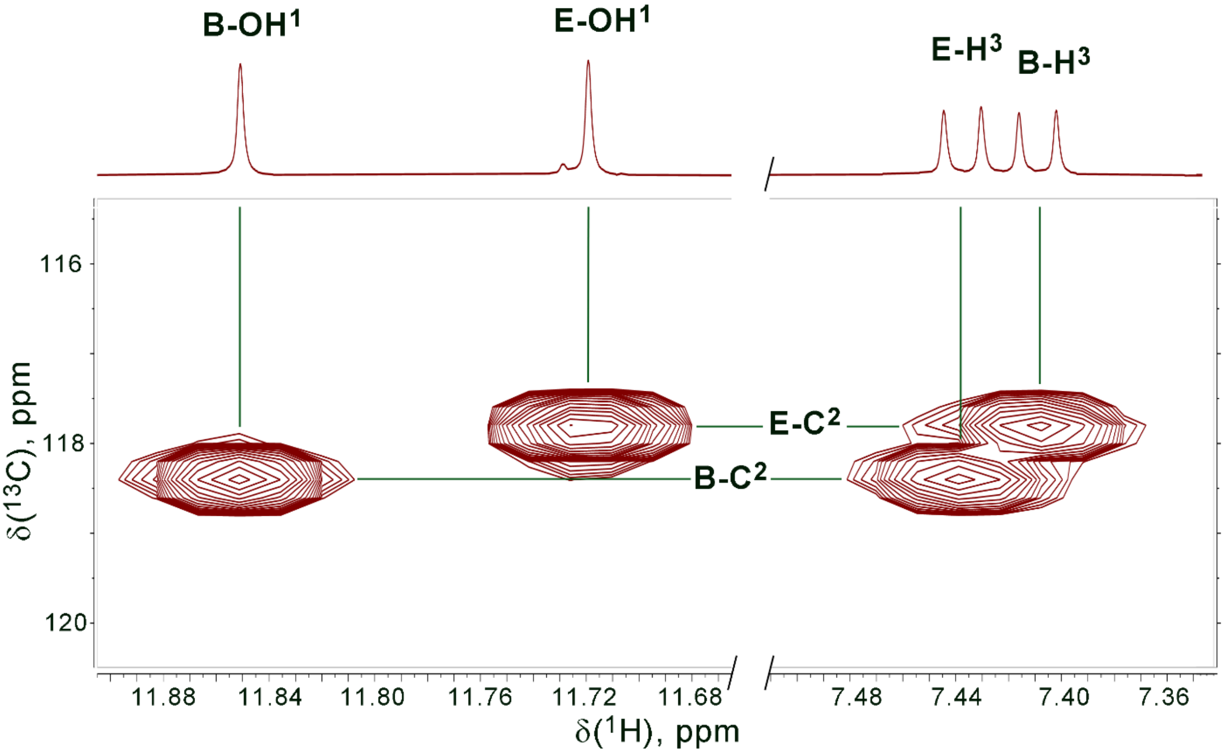

Figure S13. Fragment of the 2D ROESY spectrum of the compound **2** (secalonic acid F, composed of hemisecalonic units E and B) recorded in $CDCl_3$ at 600 MHz and 298K. Correlations between H3 and OH1 nuclei from different (E and B) hemisecalonic units are shown.

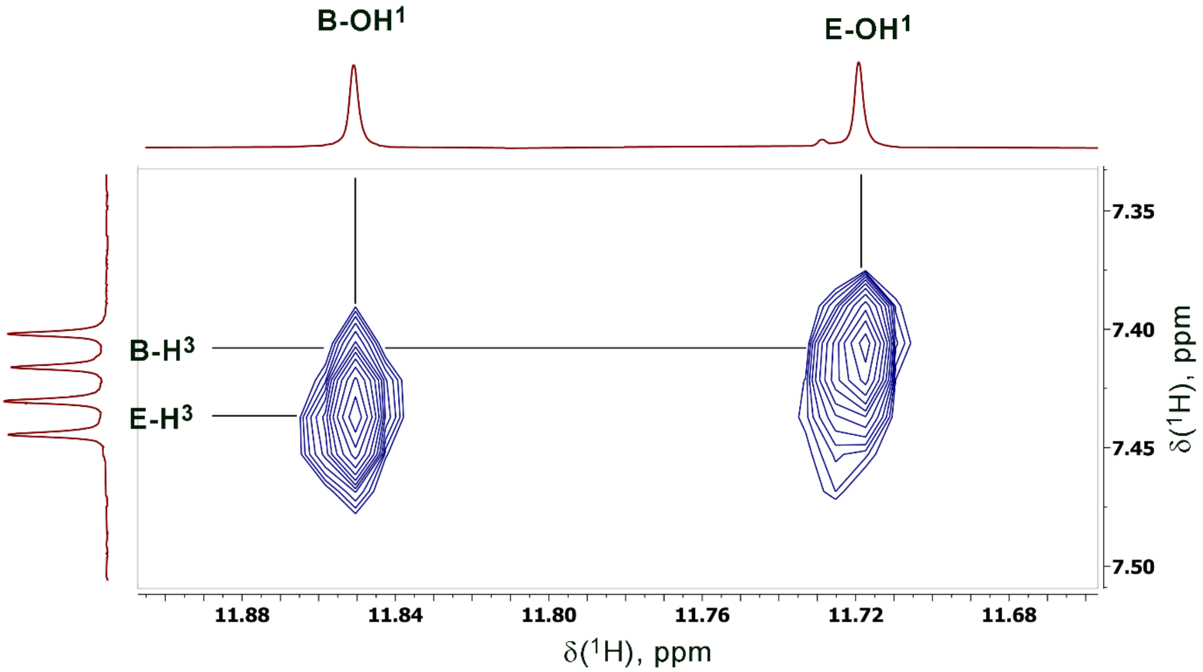

Figure S14. Validation of the stereochemistry of hemisecalonic acid units by analyzing ^3J coupling constants in NMR spectra. The ^1H-^1H coupling constant $^3J(H_5,H_6)$ was measured from 1D ^1H spectra, while heteronuclear ^1H-^{13}C coupling constants were inferred from cross-peak intensities in 2D HMBC spectra.

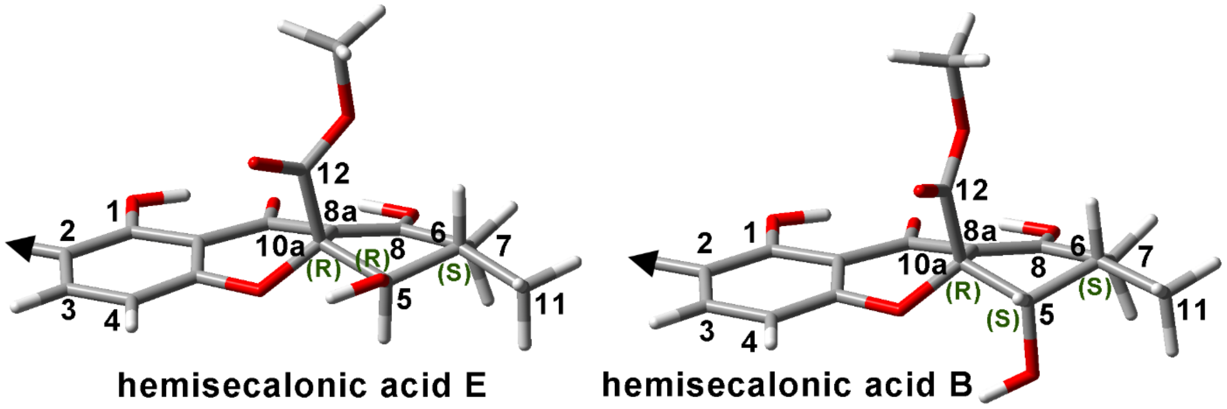

Coupling constant	Hemisecalonic acid E	Hemisecalonic acid B
$^3J(H_5,C_{8a})$	small	large
$^3J(H_5,C_{12})$	large	small
$^3J(H_5,H_6)$	11.2 Hz	1.7 Hz
$^3J(H_5,C_7)$	small	large
$^3J(H_6,C_{10a})$	small	small

Table S4. ^{1}H and ^{13}C chemical shifts of secalonic acids D and F measured in $CDCl_3$ and DMSO-d_6. Literature values are highlighted in cyan. The abbreviations hsa-E and hsa-B represent the hemisecalonic acid units E and B.

Atoms	**Secalonic acid D**				**Secalonic acid F**			
	hsa-E	hsa-E(1)	hsa-E	hsa-E(1)	hsa-E	hsa-B	hsa-E(2)	hsa-B(2)
	$CDCl_3$	$CDCl_3$	DMSO-d_6	DMSO-d_6	$CDCl_3$		$CDCl_3$	
C1	159.40	159.4	158.62	158.9	159.38	159.45		
C2	118.25	118.2	117.35	117.3	118.22	118.78		
C3	140.20	140.2	140.26	140.2	140.19	139.74		
C4	107.61	107.6	107.56	107.5	107.64	107.59		
C4a	158.26	158.3	158.99	158.5	158.26	157.20		
C5	77.03		75.31	75.2	77.01	71.42		
C6	29.24	29.2	30.02	29.9	29.28	28.50		
C7	36.30	36.2	35.96	35.8	36.31	32.66		
C8	177.52	177.5	178.26	178.2	177.56	179.85		
C8a	101.54	101.5	101.82	101.7	101.50	99.94		
C9	187.17	187.1	186.59	186.6	187.14	187.57		
C9a	106.89	106.8	106.42	106.3	106.92	107.03		
C10a	84.78	84.7	85.28	85.2	84.77	84.83		
C11	18.00	18.0	17.88	17.8	18.06	17.56		
C12	170.27	170.3	170.06	170.0	170.26	171.25		
C13	53.28	53.3	52.96	52.6	53.52	53.34		
OH1	11.73	11.76	11.60	11.64	11.72	11.85	11.65	11.80
H3	7.44	7.46	7.45	7.46	7.44	7.41	7.35	7.39
H4	6.61	6.63	6.62	6.63	6.61	6.56	6.52	6.58
H5	3.91	3.93	3.80	3.81	3.91	4.11	3.87	4.09
OH5			6.02	6.04				
H6	2.40	2.41	2.30	2.30	2.40	2.10		
H7a	2.30	2.32	2.47	2.48	2.30	2.38		
H7b	2.72	2.74	2.65	2.66	2.72	2.51	2.86	2.67
OH8	13.76	13.78	13.59	13.60	13.76	13.95	13.70	13.88
H11	1.15	1.17	1.03	1.03	1.16	1.16	1.14	1.14
H13	3.71	3.73	3.60	3.61	3.71	3.71	3.67	3.67

(1) Qin, T., & Porco Jr, J.A. (2014). Total syntheses of secalonic acids A and D. *Angewandte Chemie Int. Ed.*, ***126***(12), 3171-3174. doi: 10.1002/anie.201311260.

(2) Andersen, R., Buechi, G., Kobbe, B., & Demain, A. L. (1977). Secalonic acids D and F are toxic metabolites of *Aspergillus aculeatus*. *The Journal of Organic Chemistry*, ***42***(2), 352-353. doi: 10.1021/jo00422a042.
